# An exhaustive multiple knockout approach to understanding cell wall hydrolase function in *Bacillus subtilis*

**DOI:** 10.1128/mbio.01760-23

**Published:** 2023-09-28

**Authors:** Sean A. Wilson, Raveen K. J. Tank, Jamie K. Hobbs, Simon J. Foster, Ethan C. Garner

**Affiliations:** 1 Department of Molecular and Cellular Biology, Harvard University, Cambridge, Massachusetts, USA; 2 Center for Systems Biology, Harvard University, Cambridge, Massachusetts, USA; 3 Department of Physics and Astronomy, University of Sheffield, Sheffield, United Kingdom; 4 School of Biosciences, University of Sheffield, Sheffield, United Kingdom; Fred Hutchinson Cancer Center, Seattle, Washington, USA

**Keywords:** peptidoglycan hydrolases, *Bacillus subtilis*, genetics, cwlO, lytE, cell wall, peptidoglycan

## Abstract

Most bacteria are surrounded by their cell wall, containing a highly cross-linked protective envelope of peptidoglycan. To grow, bacteria must continuously remodel their wall, inserting new material and breaking old bonds. Bond cleavage is performed by cell wall hydrolases, allowing the wall to expand. Understanding the functions of individual hydrolases has been impeded by their redundancy: single knockouts usually present no phenotype. We used an exhaustive multiple-knockout approach to determine the minimal set of hydrolases required for growth in *Bacillus subtilis*. We identified 42 candidate hydrolases. Strikingly, we were able to remove all but two of these genes in a single strain; this “∆40” strain shows only a mild reduction in growth rate, indicating that none of the 40 hydrolases are necessary for growth. The ∆40 strain does not detectably shed old wall, suggesting that turnover is not essential for growth. The remaining hydrolases in the ∆40 strain are LytE and CwlO, previously shown to be synthetically lethal. Either can be removed in ∆40, indicating that either hydrolase alone is sufficient for cell growth. Screening of environmental conditions and biochemistry revealed that LytE activity is inhibited by Mg^2+^ and that RlpA-like proteins may stimulate LytE activity. Together, these results suggest that the only essential function of cell wall hydrolases in *B*. *subtilis* is to enable cell growth by expanding the wall and that LytE or CwlO alone are sufficient for this function. These experiments introduce the ∆40 strain as a tool to study hydrolase activity and regulation in *B. subtilis*.

**IMPORTANCE:**

In order to grow, bacterial cells must both create and break down their cell wall. The enzymes that are responsible for these processes are the target of some of our best antibiotics. Our understanding of the proteins that break down the wall— cell wall hydrolases—has been limited by redundancy among the large number of hydrolases many bacteria contain. To solve this problem, we identified 42 cell wall hydrolases in *Bacillus subtilis* and created a strain lacking 40 of them. We show that cells can survive using only a single cell wall hydrolase; this means that to understand the growth of *B. subtilis* in standard laboratory conditions, it is only necessary to study a very limited number of proteins, simplifying the problem substantially. We additionally show that the ∆40 strain is a research tool to characterize hydrolases, using it to identify three “helper” hydrolases that act in certain stress conditions.

## INTRODUCTION

Most bacterial cells are surrounded by a peptidoglycan (PG) cell wall—a load-bearing structure that protects cells from lysing due to their high internal turgor (1). Bonds must be broken in the PG for the cells to expand during growth (2). PG is built from disaccharide subunits linked to stem peptides. As new PG is inserted into the wall, the disaccharides are polymerized into long chains, and their stem peptides are cross-linked into the existing wall (3).

The enzymes that break PG bonds are termed cell wall hydrolases (hereafter “hydrolases”). Hydrolases fall into several broad categories with different chemical specificities (4). Amidases cleave the stem peptide from the sugar subunit. Endopeptidases cleave bonds between peptides within the stem peptide. Lytic transglycosylases (LTGs) and lysozymes (both of which are muramidases), cleave between the disaccharide subunits (MurNAc-GlcNAc), reversing the transglycosylase reaction that polymerizes glycan chains. Glucosaminidases target the other bond between sugar subunits (GlcNAc-MurNAc), reversing a cytoplasmic step of PG synthesis. The LTG reaction mechanism is not a hydrolysis reaction; however, to avoid introducing new terminology and improve readability, we will generally use the term “hydrolases” to refer to all PG cleavage enzymes, including LTGs. A wide array of different protein domains are capable of hydrolase activity—for example, there are at least seven distinct domains with LTG activity and well over 100 distinct domains with hydrolase activity discovered thus far (5, 6).

Hydrolase activity is essential: without the breakage of PG bonds, the cell wall cannot expand to accommodate the accumulating biomass it contains (2). Hydrolases are also involved in a variety of other processes that require modification of the cell wall: turning over old PG, cell separation, sporulation, conjugation, and motility (4, 7). Perhaps owing to the multiple cellular functions that require hydrolases, many bacteria have a large number of hydrolases. *Bacillus subtilis* and *Escherichia coli,* for example, each contain at least 20 hydrolases (4, 8). The large number of hydrolases in each bacterium, combined with a high degree of functional and enzymatic redundancy between them, has made it difficult to identify specific cellular functions for many hydrolases. Single knockouts rarely present clear phenotypes due to compensation by other hydrolases (4, 9). However, multiple-knockout approaches in *B. subtilis* have been successful in revealing the importance of LytE and CwlO for cell growth, uncovering the role of LytC and LytD in cell wall turnover, and identifying LytE, LytF, and CwlS as cell separation hydrolases (4, 10, 11).

*lytE* and *cwlO* had been previously shown to be synthetically lethal when both are deleted in *B. subtilis* (10, 12). The requirement of LytE or CwlO for cell growth was demonstrated via microscopy and genetics: upon depletion of LytE in a *cwlO* null mutant, or vice versa, cell elongation slows and then stops completely before cells lyse (12). To test whether any other hydrolases were essential for *B. subtilis* growth, we employed an exhaustive multiple-knockout approach. We created a minimal hydrolase strain that allows the study of hydrolases in isolation, making it easier to assign functions to uncharacterized hydrolases. Using this multiple hydrolase knockout strain, it is straightforward to assay the biochemical activity and determine the effect of hydrolases alone or in any desired combination on phenotypes like cell width, cell wall turnover, cell growth, or any other process.

## RESULTS

### Construction of a multiple hydrolase knockout strain

To identify the minimal set of hydrolases required for growth in *B. subtilis* PY79, we constructed a strain in which we sequentially removed as many hydrolases as possible. We used PHMMER to screen the *B. subtilis* proteome for proteins containing cell wall hydrolase domains present in known hydrolases [(4, 5, 8, 13); Table S2]. The results of this search are shown in Table 1. Cell wall hydrolases present in *B. subtilis* 168 but not present in PY79, our wild-type (WT) background, are included for completeness, though we did not generate knockouts for these. Candidate hydrolases previously shown to be unable to degrade intact PG (indicated in Table 1) were also not knocked out, nor were candidate hydrolases with transmembrane domains, as these are unlikely to be able to reach into the cell wall space far enough to directly participate in growth. We found 56 candidate hydrolases in the initial search (all shown in Table 1); of these, 50 were present in PY79. Four were excluded due to previous work demonstrating lack of activity against intact PG (AmiE, NagZ, LytB, and PgdS), and four were excluded due to being membrane-bound (MltG, SweC, SpoIIQ, and SpoIVFA), leaving 42 candidate hydrolases for our study.

**TABLE 1:** List of cell wall hydrolases in *Bacillus subtilis* identified using PHMMER^*a*^

Name (alias)	PY79	UniProt	Locus tag	Regulons	*e*-value	References	Activity
**AMIDASE**							
*Amidase_2 (PF01510)*							
cwlA	Y/KO	P24808	BSU25900		5.60^E-19^	(14 – 16)	Amidase (14)
cwlH (yqeE)	Y/KO	P54450	BSU25710	gerE^*, sigK^*	7.50^E-25^	(17)	Amidase (17)
xlyA	Y/KO	P39800	BSU12810	xpf*	1.00^E-23^	(18)	Amidase (19)
xlyB (yjpB)	Y/KO	O34391	BSU12460		2.20^E-18^	Similarity; xlyA	ND
blyA (yomC)	N	O31982	BSU21410		2.30^E-18^	(20)	Amidase (20)
*Amidase_3 (PF01520)*							
cwlC	Y/KO	Q06320	BSU17410	sigK^*	3.4^E-44^	(21, 22)	Amidase (22)
cwlD	Y/KO	P50864	BSU01530	lexA^*, sigE^*, sigG^*	1.10^E-48^	(14 – 16, 23, 24)	Amidase (25)
lytC (cwlB)	Y/KO	Q02114	BSU35620	sigA^*, sigD^*, sinR^*, slrR^*, yvrHB^*	6.4^E-54^	(7, 22, 26 – 28)	Amidase (28)
yqiI	Y/KO	P54525	BSU24190	sigA*	5.50^E-56^	(29)	SU (29)
yrvJ	Y/KO	O32041	BSU27580	sigH^	2.30^E-44^	Similarity; lytC	ND
*Amidase_6 (PF12671)*							
yhbB (ygaQ)	Y/KO	O31589	BSU08920	sigE^*	1.50^E-39^	Uncharacterized	ND
yjcM	Y/KO	O31635	BSU11910	abrB*, sigD*	1.30^E-23^	Uncharacterized	ND
*SpoIIP (PF07454)*							
spoIIP	Y/KO	P37968	BSU25530	sigE^*, sigF^*, sigG^*, spoVT^*	9.70^E-79^	(30, 31)	Amidase, DDEP (31)
*Beta-lactamase (PF00144)*							
pbpE	Y/KO	P32959	BSU34440	sigW^*	3.40^E-61^	(32)	SU (32)
pbpX	Y/KO	O31773	BSU16950	sigM^*, sigV^*, sigW^, sigX^*	7.90^E-54^	Similarity; pbpE	ND
amiE (ybbE)	Y	O05213	BSU01670	murR*	7.40^E-76^	Does not hydrolyze PG (33)	
**N-ACETYLGLUCOSAMIDASE/LYTIC TRANSGLYCOSYLASE**							
*Glyco_hydro_3 (PF01915)*							
nagZ (yzbA, ybbD)	Y	P40406	BSU01660	murR*	6.50^E-131^	Does not hydrolyze PG (33)	
*Glyco_hydro_18 (PF00704)*							
yaaH (sleL)	Y/KO	P37531	BSU00160	sigB^*, sigE^*, spoIIID^*	1.20^E-26^	Cleaves small fragments (34 – 36)	Glucosaminidase [*Bacillus anthracis* SleL (35)]
ydhD	Y/KO	O05495	BSU05710	sigE^*	9.60^E-30^	Similarity; yaaH	ND
ykvQ	Y/KO	O31682	BSU13790	sigK^*	2.50^E-23^	Similarity; yaaH	ND
yvbX	Y/KO	O32258	BSU34020		1.10^E-33^	Similarity; yaaH	ND
*Glucosaminidase (PF01832)*							
lytD (cwlG)	Y/KO	P39848	BSU35780	sigD^*, sigG^	3.30^E-11^	(7, 37, 38)	Glucosaminidase (37)
lytG	Y/KO	O32083	BSU31120		6.80^E-24^	(39)	Glucosaminidase (39)
*3D (PF06725)*							
yabE	Y/KO	P37546	BSU00400	sigA*	1.80^E-22^	Similarity; yuiC, yocH	ND
yocH	Y/KO	O34669	BSU19210	abrB^*, sigA^*, spo0A^*, walR^*	3.50^E-22^	(40)	SU (40)
yuiC	Y/KO	O32108	BSU32070	codY^*, sigF^*	9.40^E-21^	(41)	LTG (41)
yorM	N	O31901	BSU20330		7.90^E-11^	Similarity; yuiC, yocH	ND
*Hydrolase_2 (PF07486)*							
cwlJ (ycbQ)	Y/KO	P42249	BSU02600	sigE^*, sigK^*, spoIIID^*	1.60^E-18^	(42)	ND
sleB (ypeA)	Y/KO	P50739	BSU22930	sigG^*	1.20^E-25^	(43 – 45)	LTG (45)
ykvT	Y/KO	O31685	BSU13820	walR*	5.80^E-28^	Similarity; sleB, cwlJ	ND
*SLT (PF01464)*							
xkdO	Y/KO	P54334	BSU12680	xpf*	6.20^E-20^	Similarity; cwlQ, cwlP	ND
cwlQ (yjbJ)	Y/KO	O31608	BSU11570	sigD^*	1.70^E-34^	(46, 47)	LTG + muramidase (46)
yqbO	Y/KO	P45931	BSU26030		5.70^E-24^	Similarity; cwlQ, cwlP	ND
cwlP (yomI)	Y/KO	O31976	BSU21350		1.80^E-32^	(48)	Muramidase^*b*^ (48)
*Lysozyme_like (PF13702)*							
yocA	Y/KO	O34636	BSU19130		4.30^E-59^	Similarity; CwlT	ND
cwlT (yddH)	N	P96645	BSU04970	immR*	2.10^E-42^	(49)	Muramidase^*b*^ (49)
*DPBB_1 (PF03330)*							
ydjM (yzvA)	Y/KO	P40775	BSU06250	phoP*, walR*	1.00^E-09^	Similarity; PaRlpA (50)	ND
yoaJ (EXLX1)	N	O34918	BSU18630	fur*	2.90^E-07^	Does not hydrolyze PG (51)	
*SpoIID (PF08486)*							
spoIID (spoIIC)	Y/KO	P07372	BSU36750	sigE^*, spoIIID^*	1.30^E-23^	(31, 52)	LTG (31)
lytB (cwbA)	Y	Q02113	BSU35630	sigA^*, sigD^*, sinR^*, slrR^*, yvrHB^*	3.90^E-23^	Does not hydrolyze PG (53, 54)	
*YceG (PF02618)*							
mltG (yrrL)	Y	O34758	BSU27370	spo0A^*	3.20^E-90^	Similarity; EcMltG (55); membrane bound	LTG (56, 57)
sweC (yqzC)	Y	O32023	BSU24940	spo0A^*	1.40^E-07^	Similarity; EcMltG (55); membrane bound	ND
**PEPTIDASE**							
**DL-endopeptidase**							
*NLPC/P60 (PF00877)*							
cwlO (yzkA, yvcE)	Y	P40767	BSU34800	sigA*, walR*	4.40^E-29^	(58)	DLEP (58)
cwlS (yojL)	Y/KO	O31852	BSU19410	abh^*, abrB^*, ccpA^*, sigD^*, sigH^*	4.70^E-29^	(11)	DLEP (11)
lytE (papQ, cwlF)	Y	P54421	BSU09420	sigA^*, sigH^*, sigI^*, spo0A^*, walR^*	3.30^E-28^	(59, 60)	SU (59)
lytF (cwlE, ydhD)	Y/KO	O07532	BSU09370	sigD^*, sinR^*, slrR^*	1.40^E-28^	(61, 62)	DLEP (62)
pgdS (ywtD)	Y	P96740	BSU35860	sigD^*	1.00^E-22^	Does not hydrolyze PG (63, 64)	
ykfC	Y/KO	O35010	BSU12990	codY*	1.80^E-29^	(65)	DLEP (65)
cwlT (yddH)	N	P96645	BSU04970	immR*	3.50^E-34^	(49)	DLEP^*b*^ (49)
*Peptidase_M14 (PF00246)*							
yqgT	Y/KO	P54497	BSU24830		2.70^E-27^	Similarity to *Bacillus sphaericus* EP1 (66)	ND
*Peptidase_C92 (PF05708)*							
yycO	Y/KO	Q45607	BSU40280	sigK^*	5.0^E-5^		ND
**LD-endopeptidase**							
*Peptidase_M15 (PF01427)*							
cwlK (ycdD)	Y/KO	O34360	BSU02810		3.10^E-20^	(67)	LDEP (67)
*Peptidase_M23 (PF01551)*							
lytH (yunA, yutA)	Y/KO	O32130	BSU32340	sigK^*	1.80^E-21^	(68)	LDEP (68)
spoIIQ	Y	P71044	BSU36550	sigF^*	1.00^E-24^	Membrane bound	ND
spoIVFA	Y	P26936	BSU27980	sigE^*, spoIIID^*	3.40^E-12^	Membrane bound	ND
cwlP (yomI)	N	O31976	BSU21350		1.80^E-27^	(48)	DDEP^*b*^ (48)

^*a*^ Cell wall hydrolases were identified via a PHMMER (13) search with default parameters of the *B. subtilis* subsp. 168 and *B. subtilis* subsp. PY79 proteome for PFAM domains associated with known cell wall hydrolases (Table S2). For each hydrolase, we report its name (and any aliases), whether it is knocked out in the ∆40 strain (KO) or present in PY79 (Y/N), its UniProt accession number, its locus tag, any reported regulons it is a member of [^ indicates source Faria et al. 2016 (69), * indicates source SubtiWiki (70)], the PHMMER search significance *e*-value, and any relevant references showing its biochemical activity. DLEP, D,L-endopeptidase; LDEP, L,D-endopeptidase; DDEP, D,D-endopeptidase; LTG, lytic transglycosylase; SU, specificity untested (only cell wall degradative activity shown); ND, no data.

^*b*^ CwlP and CwlT are two-domain cell wall hydrolases and so appear twice in this table. Only the activity for the specific PFAM domain is listed in the Activity column.

**Fig 1:**
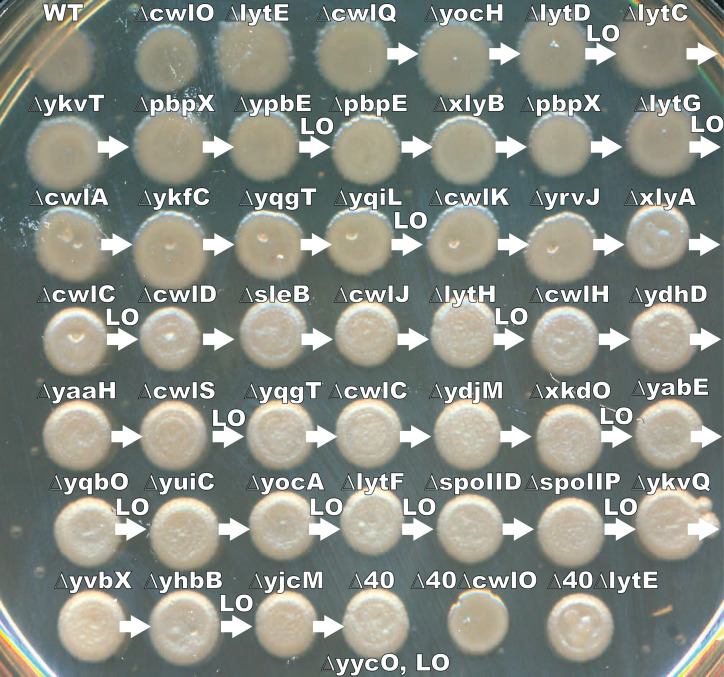
Construction of the ∆40 strain via sequential knockout and loopout. Colony morphology of each cloning intermediate for the ∆40 strain. WT cells were transformed with a series of resistance-cassette-marked knockouts (starting with *∆cwlQ*). Periodically, antibiotic-resistance cassettes were removed via Cre-loxP mediated loopout (indicated by LO). Arrows indicate sequential integrations (e.g., the strain indicated by *∆yocH* contains *∆yocH* and *∆cwlQ*). Dense cell suspensions were spotted and incubated overnight to visualize colony morphology.

We next generated single knockouts for each of the candidate hydrolases by replacing the gene with an antibiotic resistance cassette flanked by loxP sites. We then sequentially combined all knockouts into a single strain, using Cre-lox mediated loopouts to remove markers when necessary (Fig. 1). After each loop-out step, we verified the deletion of all modified loci by PCR. After all knockouts had been combined into a single strain, whole-genome sequencing was used to confirm all deletions and to identify any genomic rearrangements or mutations that could have occurred during the construction process. Despite the multiple rounds of transformation and loopouts this strain was subjected to, with up to four resistance cassettes removed simultaneously multiple times, we found no evidence of genomic rearrangements based on read coverage from the DNA extracted during exponentially growing cells (Fig. S1) (71), and only eight SNPs leading to five point mutations in genes involved in unrelated processes (Table S1).

Ultimately, this effort produced a strain lacking 40 hydrolases, which we termed “∆40.” The ∆40 strain is lacking all the identified hydrolases that met our criteria save two—LytE and CwlO, two synthetically lethal endopeptidases previously shown to be essential for growth (10). We were able to further knock out either *lytE* or *cwlO* in the ∆40 strain, but not both, due to their synthetic lethality.

### Hydrolase activity is greatly reduced in the ∆40 strain

**Fig 2:**
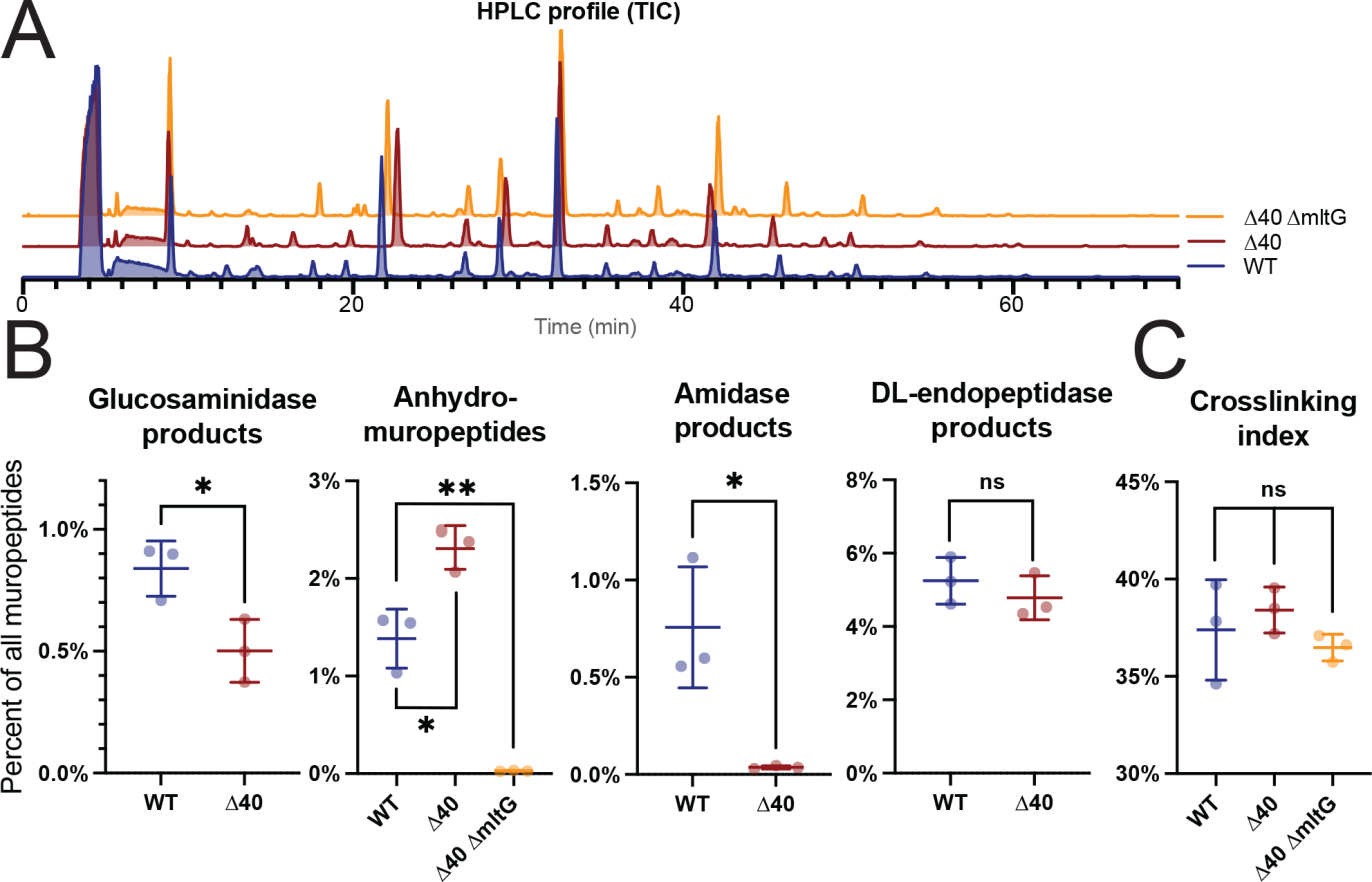
The ∆40 strain has a reduced cell wall hydrolytic complement. (A) HPLC analysis of isolated muropeptides in WT and ∆40 strains. Purified cell walls were digested to yield soluble muropeptides, which were separated and characterized via HPLC-MS (high-performance liquid chromatography followed by mass spectrometry). The total ion current (TIC) elution profile is shown. Strains used: PY79, WT; bSW431, ∆40; and bSW537, ∆40 *∆mltG*. (B) Identification of cell wall hydrolase products in WT and ∆40 cells by peptidoglycan profiling. High-resolution mass spectrometry was used to identify separated muropeptides. Muropeptides missing a GlcNAc were classified as glucosaminidase products. Anhydromuropeptides were classified as LTG products. Cross-linked muropeptides lacking MurNAc-GlcNAc and MurNAc-GlcNAc itself were classified as amidase products. Cross-linked muropeptides lacking MurNAc-GlcNAc-L-Ala-iso-D-Glu, and MurNAc-GlcNAc-L-Ala-iso-D-Glu itself, were classified as D,L-endopeptidase products. For additional details about muropeptide classification, see Table S3. The cross-linking index was calculated as in reference (73). For each set of hydrolase products, the sum of the MS intensity for those products was divided by the total MS intensity for all detected muropeptides (percentage of total). D,L-endopeptidase products are still present as expected because the strain retains the D,L-endopeptidases LytE and CwlO. Amidase products are strongly reduced. Glucosaminidase products are reduced in abundance by roughly twofold. Lytic transglycosylase products are still present in the ∆40 strain but are strongly reduced if *mltG* is additionally knocked out. Strains used: PY79, WT; bSW431, ∆40; and bSW537, ∆40 *∆mltG*.

To assess whether any other unidentified hydrolases remained in the ∆40 strain, we conducted PG profiling of both WT cells and the ∆40 strain (72), allowing us to determine the abundance of hydrolase products in their cell walls (Fig. 2; Table S3).

Our PG profiling assay has limitations: as PG profiling relies on muramidase digestion to yield soluble muropeptides for HPLC (high-performance liquid chromatography) analysis, we could not use this assay to detect hydrolases with muramidase activity. Likewise, as D,D-endopeptidases produce products that are indistinguishable from unmodified PG, we cannot unambiguously assign specific PG products to D,D-endopeptidases in these experiments.

We compared the relative abundance of different PG hydrolase products in the ∆40 and WT strains (Fig. 2B; Table S3). The ∆40 strain showed a very small amount of amidase activity (~20-fold reduction vs WT, 0.8% vs 0.04% of all muropeptides, *P* = 0.0161, unpaired *t*-test) and a reduction of glucosaminidase activity (approximate twofold reduction vs WT, 0.8% vs 0.5% of all muropeptides, *P* = 0.0279, unpaired *t*-test), indicating that these classes of hydrolases had been successfully reduced in the ∆40 strain. The residual glucosaminidase activity could represent (i) a yet unknown minor glucosaminidase with a novel fold or (ii) sample degradation during PG purification. We observed no change in D,L-endopeptidase activity in the ∆40 strain (5.2% vs 4.8% of all muropeptides, *P* = 0.4094, unpaired *t*-test), as expected given that ∆40 retains the D,L-endopeptidases LytE and CwlO. In agreement with previous work (72), L,D-endopeptidase activity was not detected in any strain. D,L-endopeptidases cleave between the mDap and iso-D-Glu residues in the stem peptide, while L,D-endopeptidases cleave between iso-D-Glu and L-Ala.

Unexpectedly, the ∆40 strain also showed an increase in LTG activity (Fig. 2B, ~1.75-fold increase vs WT, 1.4% vs 2.3% of all muropeptides, *P* = 0.0125, unpaired *t*-test). We found that this remaining LTG activity required MltG. Removing *mltG* from the ∆40 strain substantially reduced apparent LTG activity (Fig. 2B, ~80-fold reduction vs ∆40, 2.3% vs 0.03% of all muropeptides, *P* < 0.0001, unpaired *t-*test). MltG’s catalytic domain is predicted to be extracellular, although MltG is likely too small to reach far enough into the cell wall space to directly participate in cell wall expansion. MltG has been shown to be involved in membrane-proximal PG metabolism, cleaving PG at a specific distance from the membrane to produce 7-disaccharide long glycan strands (55 – 57).

Additionally, we measured the rate of autolysis in the ∆40 strain. Autolysis occurs when hydrolases become dysregulated and degrade the wall in an uncontrolled way, leading to cell lysis. Disruption of the energized membrane via energetic poisons or treatment with antibiotics can cause autolysis (23). In *B. subtilis*, the hydrolases LytC and LytD are the main effectors of autolysis, with LytE and LytF additionally having smaller effects (7, 61).

**Fig 3:**
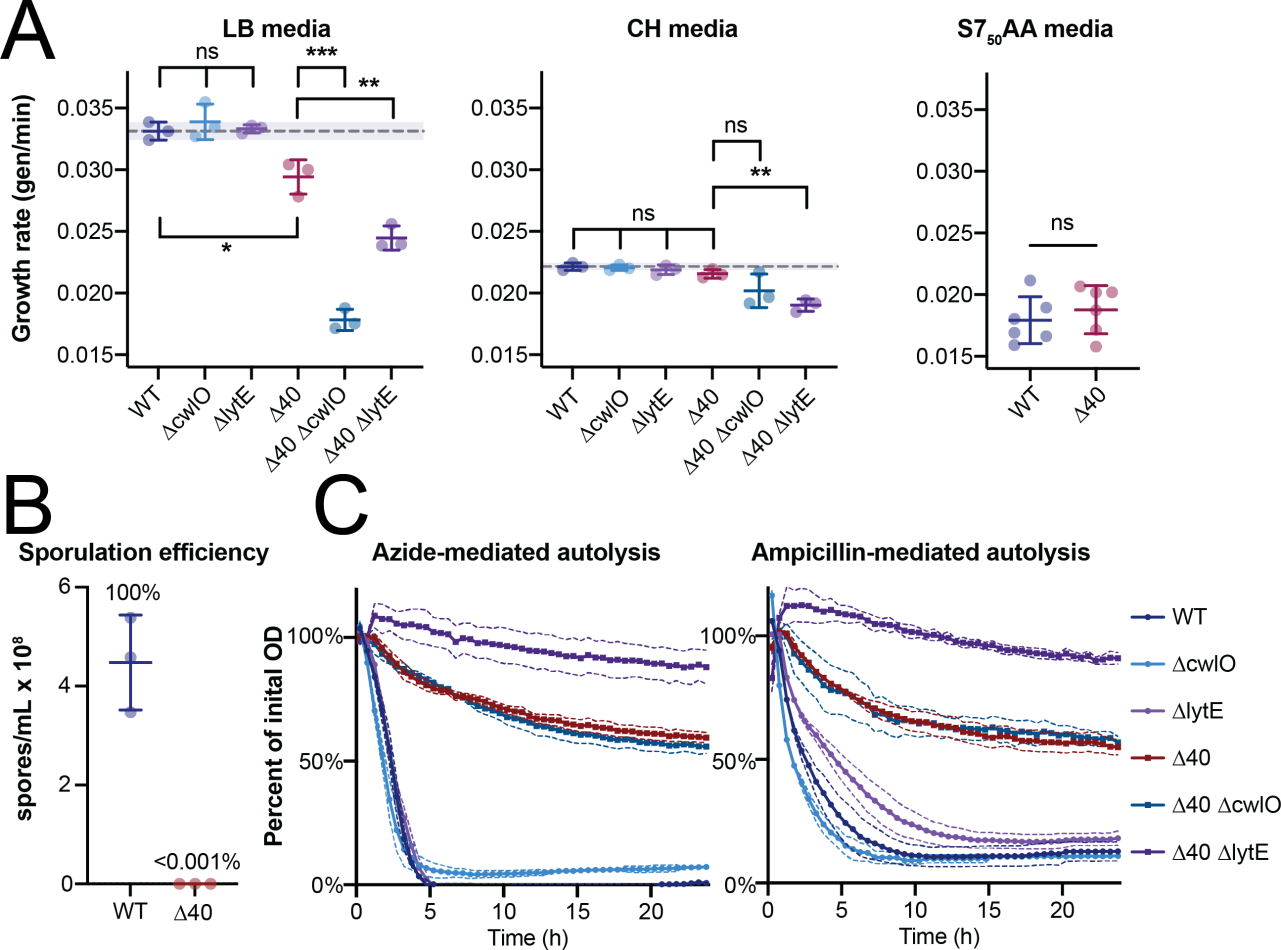
The ∆40 strain grows at a similar rate as WT, does not sporulate, and has a much slower autolysis rate in response to both sodium azide and ampicillin. (A) (left) The ∆40 strain has a slower growth rate vs WT in LB media. Cultures were grown in LB media at 37 ˚C to an OD_600_ of ~0.3–0.5, diluted to an OD_600_ of 0.05, and samples were collected every 6 min for 1 h (~3 doublings). OD_600_ vs time plots were fit to a single exponential to obtain the growth rate. Each point represents the doubling time from a single experiment, and solid lines show the mean and standard deviation. The dotted line shows the mean WT growth rate, for comparison. ∆40 has a slower growth rate to WT. *lytE* and *cwlO* knockouts grow much more slowly in the ∆40 background than in a WT background. Strains used: PY79, WT; bSW23, ∆*cwlO*; bSW295, ∆*lytE*; bSW431, ∆40; bSW433, ∆40 ∆*cwlO*; and bSW435, ∆40 ∆*lytE*. (A) (middle) The ∆40 strain has a similar growth rate to WT in CH media. Cultures were grown in CH media at 37˚C. Samples were collected and data were analyzed as in panel (A). While ∆40 has a similar growth rate to WT, *lytE* and *cwlO* knockouts grow more slowly in this background than in a WT background. Strains used: PY79, WT; bSW23, ∆*cwlO*; bSW295, ∆*lytE*; bSW431, ∆40; bSW433, ∆40 ∆*cwlO*; and bSW435, ∆40 ∆*lytE*. (A) (right) The ∆40 strain has a similar growth rate to WT in minimal media. Cultures were grown in S7_50_AA media at 37°C. Samples were collected and data were analyzed as in panel (A). Strains used: PY79, WT and bSW431, ∆40. (B) The ∆40 strain is unable to sporulate. Sporulation was induced by resuspension and sporulation efficiency was determined as in reference (30). The WT strain produced ~10^8^ spores/mL, while the ∆40 strain produced ~100 spores/mL, all of which lacked the distinctive ∆40 colony morphology upon outgrowth and likely represent contamination. Strains used: PY79, WT and bSW431, ∆40. (C) The ∆40 strain shows slower autolysis in response to sodium azide (left) and ampicillin (right). Cultures were grown in CH media at 37°C to an OD_600_ of 0.5 (azide treatment) or 0.15 (ampicillin treatment) in baffled flasks with vigorous shaking, then diluted to an OD_600_ of 0.025 in a 150 µL of prewarmed CH in a 96-well plate. Sodium azide (75 mM) or ampicillin (100 µg/mL) was added and OD_600_ readings were taken using a plate reader every 2 min for 24 h. The plate was shaken vigorously in between the measurements. The ∆40 strain takes around 10 times as long to reach half the initial OD_600_ as the WT strain, and the ∆40 ∆lytE strain, in particular, has a strongly reduced rate of autolysis. Strains used: PY79, WT; bSW23, ∆*cwlO*; bSW295, ∆*lytE*; bSW431, ∆40; bSW433, ∆40 ∆*cwlO*; and bSW435, ∆40 ∆*lytE*.

*∆cwlO* and *∆lytE* cells had approximately WT rates of autolysis, with nearly 100% of cells being lysed after 5 h of treatment with 75 mM sodium azide or 100 µg/mL ampicillin (Fig. 3C). In contrast, the ∆40 strain had a significantly slower rate of autolysis in both treatment conditions, with the ∆40 *∆lytE* strain showing only a ~10% reduction in OD_600_ after 24 h of treatment and the ∆40 strain itself showing a ~40% OD_600_ reduction after 24 h (Fig. 3C). The non-zero autolysis rate in the ∆40 *∆lytE* background could imply the involvement of CwlO in autolysis, could represent non-hydrolase-mediated lysis, or could suggest a remaining hydrolase with a minor role in autolysis.

### Cell growth and morphology are similar in the ∆40 strain relative to wild type

**Fig 4:**
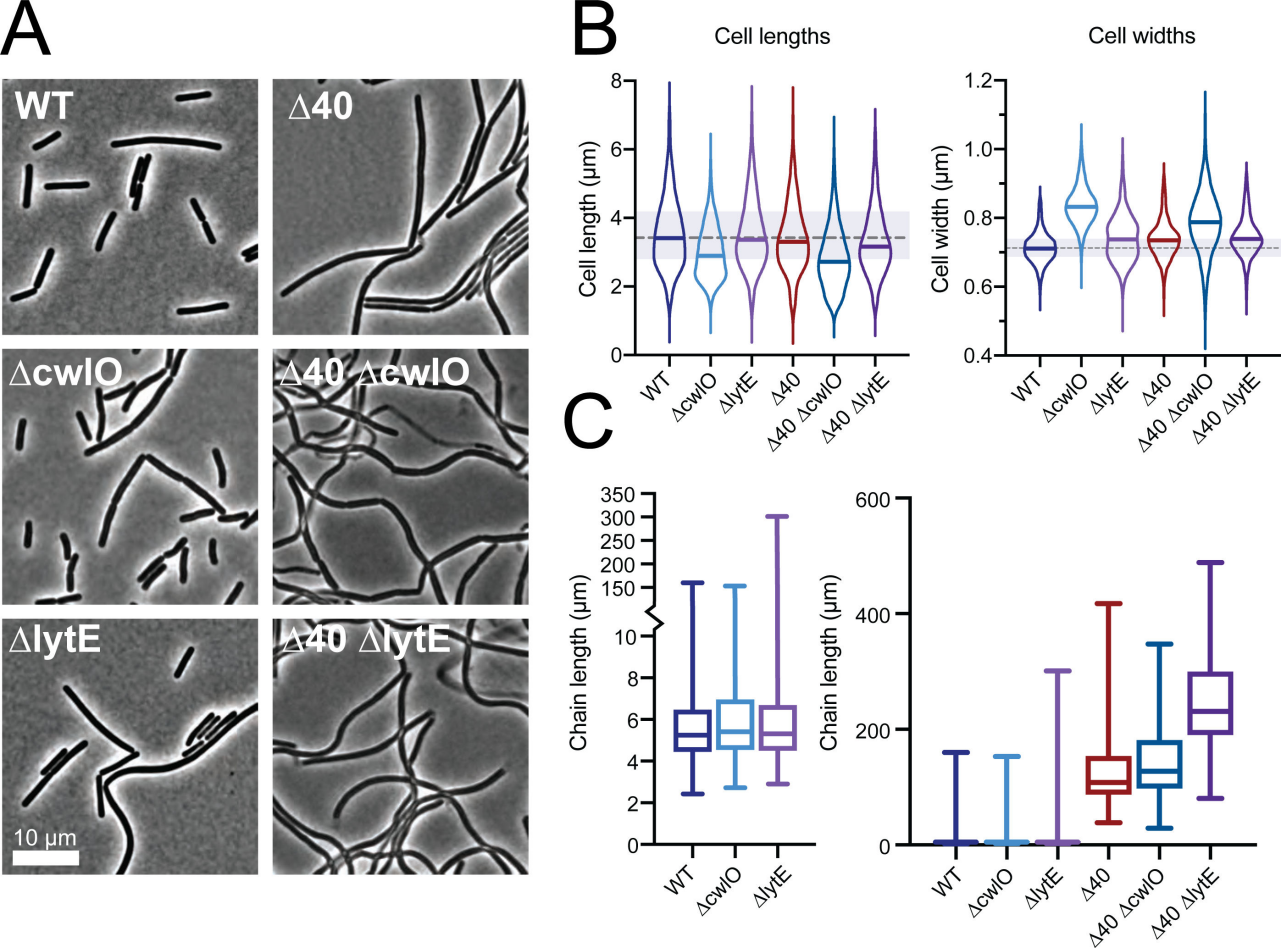
The ∆40 strain has mild shape defects but a significant chaining phenotype. (A) Representative phase contrast images of hydrolase mutant strains. ∆40 cells primarily form long chains, ∆40 ∆*cwlO* cells have variable widths, and ∆40 ∆*lytE* cells sometimes have phase-light, lysed cells still attached to their poles (see Fig. S2 for TEM images). Both ∆40 *∆cwlO* and ∆40 *∆lytE* have a population of phase-light, lysed cells. Scale bar is 10 µm. (B) Cell lengths (left) and widths (right) in hydrolase mutants. Cells were labeled with membrane stain and imaged by epifluorescence microscopy. Cell dimensions were measured from these images using Morphometrics (74). Solid lines in violins show medians. Dashed line outside violins shows WT median for comparison. Shaded region outside violins shows WT quartiles. Strains used: PY79, WT; bSW23, ∆*cwlO*; bSW295, ∆*lytE*; bSW431, ∆40; bSW433, ∆40 ∆*cwlO*; and bSW435, ∆40 ∆*lytE*. (C) Chain lengths in hydrolase mutants (left, zoomed view of the right). Chain length was measured manually from tiled and stitched phase contrast images. The ∆40 strain’s median chain length is around 30 times longer than the WT median chain length. Strains used: PY79, WT; bSW23, ∆*cwlO*; bSW295, ∆*lytE*; bSW431, ∆40; bSW433, ∆40 ∆*cwlO*; and bSW435, ∆40 ∆*lytE*.

We next characterized the growth rate of the ∆40 strain. The ∆40 strain grew slightly slower than WT cells in rich, undefined media [Luria Broth (LB)], but grew at the same rate as WT cells in both rich, defined media [casein hydrolysate (CH)] and fully synthetic media (S7_50_ with glucose and amino acids, see Materials and Methods for details) (Fig. 3A). This suggests that the activity of LytE and CwlO together is mostly sufficient for normal cell growth, although when pushed towards higher growth rates other cell wall hydrolases may contribute to growth. To investigate the individual effects of LytE and CwlO on the cell growth rate, we made knockouts of *lytE* and *cwlO* in both WT and ∆40 backgrounds. ∆40 ∆*lytE* and ∆40 ∆*cwlO* both exhibited a reduction in growth rate compared to ∆40, which was especially pronounced in LB media. We observed cell lysis in both ∆40 ∆*lytE* and ∆40 ∆*cwlO* strains in phase-contrast images, which could contribute to their slower growth rates as measured in bulk by OD_600_ (Fig. 4A). On the other hand, ∆*lytE* or ∆*cwlO* in a WT background had the same growth rate as WT. This suggests that in WT cells, other hydrolases participate in but are not strictly required for growth, or that LytE and CwlO are not being expressed highly enough to maintain normal growth on their own in the ∆40 background.

Next, we quantified cell dimensions in CH media in these strains using FM 5-95 membrane stain. ∆40 cells had a WT cell length and were 3% wider (Fig. 4B, *P* < 0.0001, unpaired *t*-test with Welch’s correction). ∆*cwlO* cells were 13% wider and 18% shorter than WT cells, a phenotype that persisted in the ∆40 ∆*cwlO* strain (Fig. 4B, *P* < 0.0001 for all comparisons: unpaired *t*-test with Welch’s correction for width comparisons, Mann-Whitney test for length comparisons). ∆40 ∆*cwlO* cells were less able to control their width as compared to ∆40 cells, having a 1.5× wider cell width distribution (Fig. 4B, 7.5% vs 11.33% coefficient of variation, *F*-test *P* < 0.001). In contrast, ∆*lytE* cells were only slightly wider than WT cells (Fig. 4B, 1%, *P* < 0.0001, unpaired *t*-test with Welch’s correction), and ∆40 ∆*lytE* cells were slightly narrower (Fig. 4B, 1%, *P* < 0.0001, unpaired *t*-test with Welch’s correction) than ∆40 strain alone, with a slight decrease in length (Fig. 4B, *P* < 0.0001, Mann-Whitney test). Thus, CwlO appears to be involved in cell-width maintenance, as removing *cwlO* causes changes in cell width both in ∆40 and WT backgrounds, consistent with previous reports (12). Furthermore, given that removing *cwlO* increases the cell width coefficient of variation in the ∆40 background but does not increase the width variation when deleted from WT cells, other hydrolases must also have a role in width homeostasis.

We then quantified the chain length for the ∆40 strain and derivatives. Individual *B*. *subtilis* cells are often found connected to their siblings via a common cell wall septum as cell separation and cell division does not always occur at the same time in this organism. Cell separation requires the action of hydrolases that cleave between the two connected cells—several different hydrolases serve this purpose in *B*. *subtilis*, primarily LytF, CwlS, and LytE (11, 59, 62). In WT cells, the average chain length was comparable to the length of individual cells (Fig. 4C, ~4.5 µm per chain vs ~3.5 µm per cell). The maximum chain length observed was 150 µm. *∆cwlO* and *∆lytE* mutants had a similar average chain length although the *∆lytE* mutant had a larger maximum chain length (Fig. 4C, 300 µm), consistent with the known role of LytE in cell separation.

In contrast, the ∆40 strain had a significant increase in the average chain length (Fig. 4C, 120 µm), nearly as long as the longest observed WT chain (150 µm). The ∆40 *∆cwlO* strain was similar to the ∆40 strain, while the ∆40 *∆lytE* strain had a large increase in the average chain length (250 µm), almost double that of the longest observed WT chain. Because the ∆40 *∆lytE* strain lacks all cell separation hydrolases, the remaining cell separation in this strain was likely due to mechanical tearing of cells under the vigorous shaking conditions needed to be able to measure accurate culture OD_600_ for these experiments; the ends of the chains had visible, phase-light debris resembling torn cells still attached visible by transmission electron microscopy (TEM) (Fig. S3). In gentler culture conditions on a roller drum, this strain grows as a large clump of cells visible to the naked eye.

Additionally, we tested the ability of the ∆40 strain to sporulate. Hydrolases are involved in both entry into sporulation and exit from the spore during germination (30, 31, 43). We found that the ∆40 was not able to sporulate (Fig. 2B, *P* = 0.3859, one sample *t*-test vs efficiency of 0), likely because it lacks SpoIID and SpoIIP, causing a block at the engulfment stage of sporulation (31).

### ∆40 cells do not detectably turn over their cell wall

**Fig 5:**
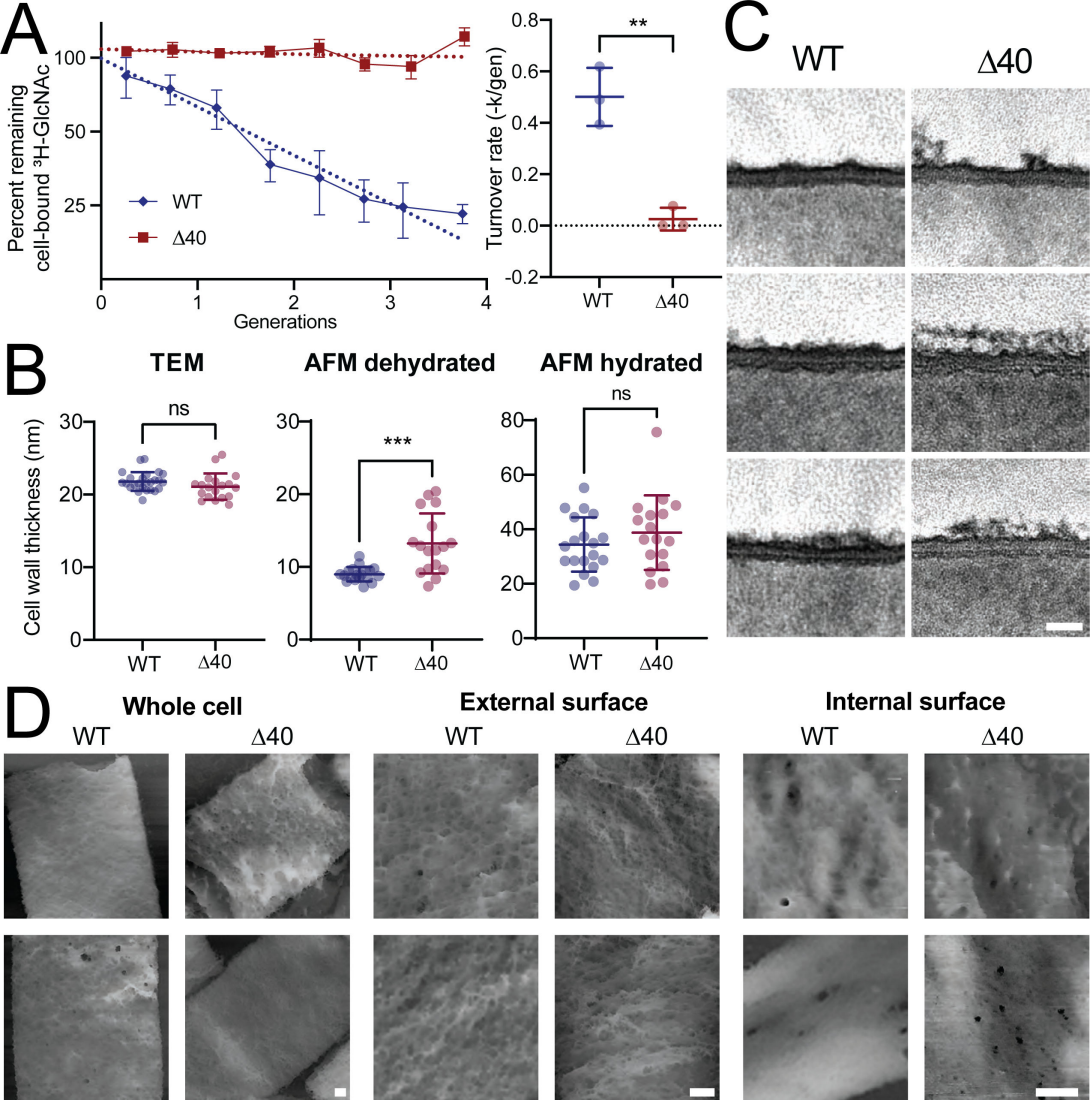
The ∆40 strain does not detectably turn over cell wall. (A) Cell wall turnover rate is negligible in the ∆40 strain. Left: pulse-chase radiolabel measurements were used to determine the cell wall turnover rate. Cells were labeled with H^3^-GlcNAc, which incorporates into the cell wall. The ^3^H-GlcNAc was then washed out and radioactivity was subsequently measured for four generations. A decrease in radioactivity indicates that material is being removed from the cell wall, i.e., that cell wall is turning over. Each experiment was replicated at least three times. Dotted lines show single exponential fit to mean data. Right: single exponential fits to each experiment at left. Each point represents the time constant (-k) obtained from a fit to a single experiment. Error bars show SD. The ∆40 turnover rate is not significantly different from zero (one sample *t*-test, *P* = 0.4837). Mean initial radioactivity was 167,766 DPM/OD_600_ for the WT strain and 174,334 DPM/OD_600_ for the ∆40 strain. Strains used: PY79, WT and bSW431, ∆40. (B) Cell wall thickness in the ∆40 strain. Cell wall thickness was measured via transmission electron microscopy and atomic force microscopy as described in Materials and Methods. Briefly, for TEM imaging, exponentially growing cells were fixed, osmicated, stained with uranyl acetate, embedded in Embed 812, sectioned, and imaged without additional staining. For AFM imaging, exponentially growing cells were boiled, broken, protease treated, and then adhered to mica for imaging. Each point is the mean cell wall thickness measured for a single cell. WT AFM measurements are reproduced from reference (76), ∆40 AFM measurements were performed as part of this study. Error bars show SD. Strains used: PY79, WT (for TEM measurements); 168, WT (for AFM measurements); and bSW431, ∆40. (C) Representative TEM images of cell wall thickness. Representative TEM images of cell wall thickness analyzed in panel (B). Strains used: PY79, WT and bSW431, ∆40. Scale bar is 20 nm. (D) Representative AFM images of sacculi. Representative AFM images of cell walls analyzed in panel (B). Strains used: 168, WT and bSW431, ∆40. Scale bars are 100 nm.

Hydrolases are involved in cell wall turnover, where old PG material is shed from the cell wall (75). We measured the rate of cell wall turnover of both WT and ∆40 cells using pulse-chase labeling with the radioactive cell wall precursor ^3^H-N-acetylglucosamine (^3^H-GlcNAc). This revealed that, while WT cells turn over PG at a rate of about 50% per generation in agreement with previous work (75), turnover in ∆40 strain was not detectable, with a rate not significantly different from zero (Fig. 5A, *P* = 0.4837, one sample *t-* test vs rate of 0). These results suggest that LytE and CwlO, the only identifiable remaining hydrolases in the ∆40 strain, likely do not contribute to cell wall turnover. Furthermore, these data suggest that cell wall turnover is not an essential process: cell growth only requires the cleavage of bonds so the cell can expand.

As hydrolase-deficient mutants have been shown to have altered cell wall thickness (77, 78), we measured the cell wall thickness of the ∆40 strain using TEM and atomic force microscopy (AFM). We found that the wall was significantly thicker only in dehydrated samples measured using AFM (Fig. 5B, center, *P* = 0.0007, unpaired *t*-test); measurements on TEM images or on hydrated AFM samples showed no significant differences (TEM: Fig. 5B, left, *P* = 0.1382, unpaired *t*-test; hydrated AFM: Fig. 5B, right, *P* = 0.2887, unpaired *t*-test). The WT cell wall was more uniform in appearance in both TEM and AFM images, while the ∆40 strain had more heterogeneity in density and thickness, particularly on the outer face of the wall, with an increase in the presence of “ruffles” on the outer face of the cell wall in the ∆40 strain (Fig. 5C and D). These “ruffles” may represent the additional old cell wall material present due to the strongly reduced turnover rate. The internal face of the cell wall appeared denser than the external face of the wall in both the WT and ∆40 strains (Fig. 5D). The internal face of the cell wall in the ∆40 strain appeared to have both a denser meshwork and an increased number of larger pores compared to the WT strain.

Substantial changes to the cell wall ultrastructure occur during sample preparation for TEM, especially in the outer layers of the cell wall (79 – 81); it would be interesting to apply additional, less perturbative EM modalities such as cryo-electron microscopy to the ∆40 strain to help clarify the exact nature of the changes to the ∆40 cell wall. It is possible that both TEM and hydrated AFM highlight mostly the denser, newer cell wall material, while dehydrated AFM allows visualization of all the cell wall, including the more loosely bound older wall material.

### ∆40 ∆*cwlO* cells are sensitive to various stresses, including ionic stress

**Fig 6:**
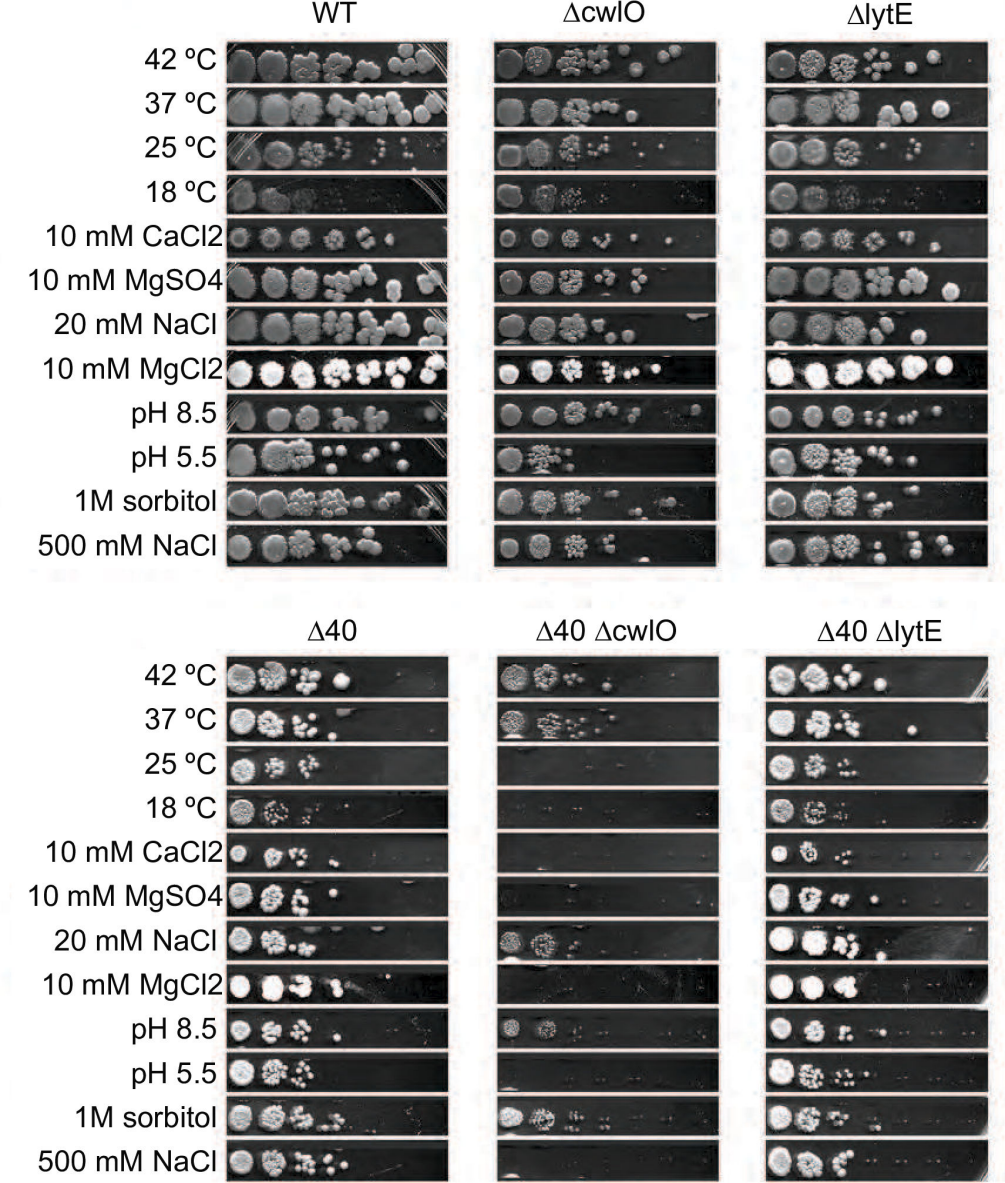
The ∆40 strain has similar viability to WT in a range of stress conditions, but ∆40 ∆*cwlO* is sensitive to ionic, cold, and low pH stress. Spot dilution assays of different strains under various stress conditions. Cultures of each strain were plated in a 1:10 dilution series onto LB plates containing various stressors and grown overnight at the specified temperature or at 37°C if not indicated. Most conditions supported normal growth, but growth of the ∆40 ∆*cwlO* strain was inhibited at 25°C, pH 5.5, or with the addition of 10 mM MgCl_2_, 10 mM MgSO_4_, 10 mM CaCl_2_, or 300 mM NaCl. Strains used: PY79, WT; bSW23, ∆*cwlO*; bSW295, ∆*lytE*; bSW431, ∆40; bSW433, ∆40 ∆*cwlO*; and bSW435, ∆40 ∆*lytE*.

Although the ∆40 strain grew mostly normally under our standard lab conditions, we wondered whether the absence of so many hydrolases would sensitize cells to stress conditions. We used a spot dilution assay to measure the viability of our strains under a variety of stress conditions: temperature, ionic stress, pH, and osmotic stress (Fig. 6). In all conditions, including our control (37˚C), ∆40 cells had fewer CFUs than WT. This is expected because ∆40 cells grow in long chains, and thus cells cannot readily separate into individual CFUs. In all stress conditions, ∆40 cells were similarly viable to WT cells, as were ∆*lytE,* ∆*cwlO,* and ∆40 ∆*lytE* cells. However, ∆40 ∆*cwlO* cells were susceptible to multiple stresses, including low pH, low temperature, and ionic stress.

**Fig 7:**
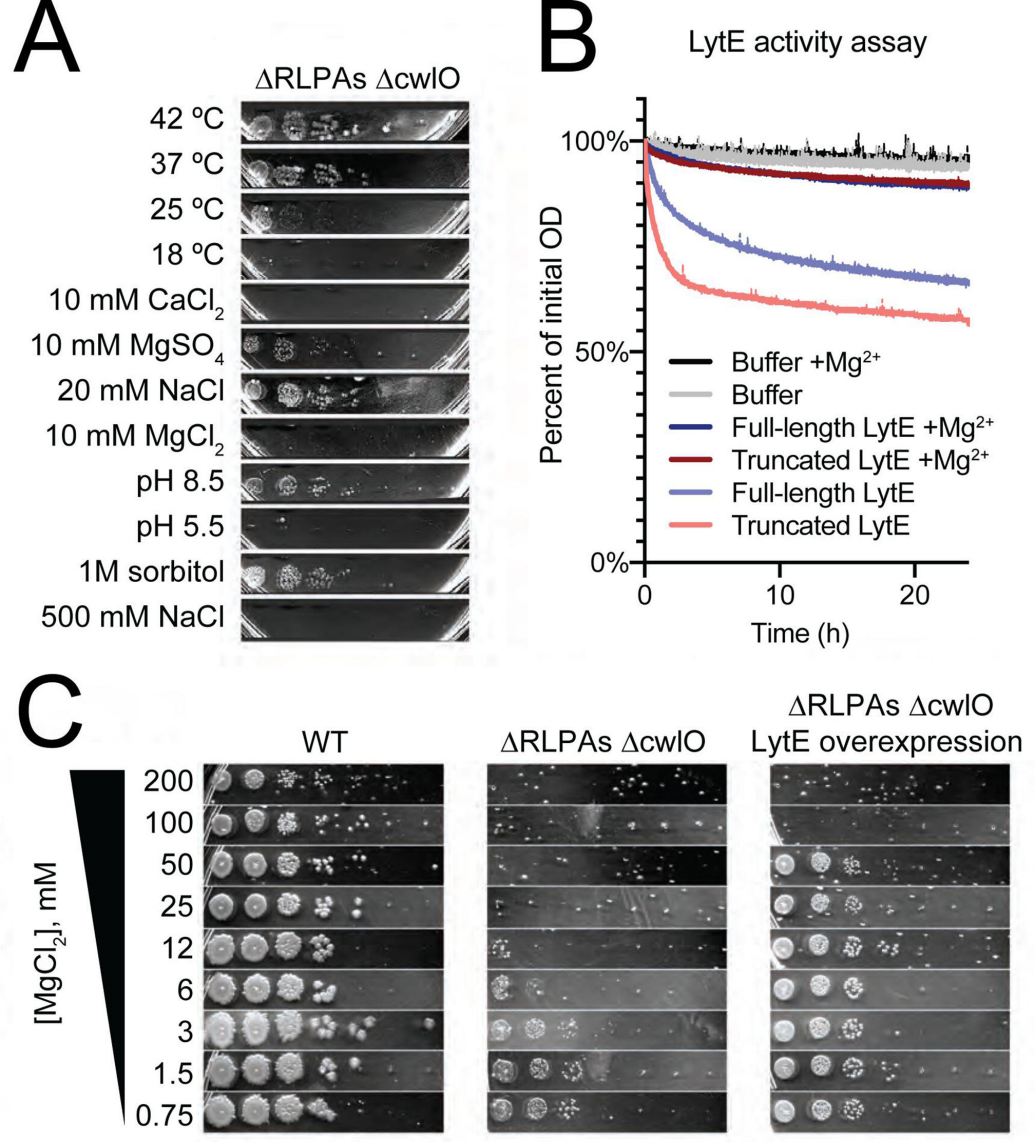
Three uncharacterized RlpA-like proteins stimulate LytE activity in the presence of divalent cations. (A) The removal of three RlpA-like proteins makes ∆*cwlO* cells stress-sensitive. Spot dilution assays were performed as in Fig. 4. ∆*yabE* ∆*yocH* ∆*ydjM* (∆RLPAs) ∆*cwlO* showed the same stress sensitivity profile as ∆40 ∆*cwlO*, except that 10 mM MgSO_4_ and 25°C only partially inhibited growth. Strain used: bSW490, ∆*cwlO* ∆*yabE* ∆*yocH* ∆*ydjM*. (B) Mg^2+^ directly inhibits LytE activity. LytE was expressed in *E. coli* and purified both with and without its N-terminal LysM domains and incubated with purified cell walls in 150 µL of 50 mM HEPES pH 7, 2 mM DTT, 500 mM NaCl with 0.5% (wt/vol) Pluronic F-108 in a 96-well plate. OD_600_ was measured every 2 min using a plate reader. The addition of 25 mM MgCl_2_ almost completely inhibited the activity of LytE. (C) LytE overexpression rescues Mg^2+^ sensitivity in the ∆RLPAs ∆*cwlO* background. Spot dilutions were performed as in Fig. 5A, with the indicated concentration of MgCl_2_ and the addition of 1 mM isopropyl β-D-thiogalactoside (IPTG) to drive LytE overexpression. Strains used: PY79, WT; (∆RLPAs) ∆*cwlO*, bSW490, ∆*cwlO* ∆*yabE* ∆*yocH* ∆*ydjM* and (∆RLPAs) ∆*cwlO* lytE overexpression, bSW519, ∆*cwlO* ∆*yabE* ∆*yocH* ∆*ydjM amyE::pHyperSpank-lytE*.

We were particularly intrigued by the susceptibility of ∆40 ∆*cwlO* to Mg^2+^. Mg^2+^ is coordinated between PG and teichoic acids (82), and this Mg^2+^ binding is thought to give structural stability to the cell wall (81, 83). High levels of Mg^2+^ are often protective against cell wall perturbations, including knockouts of hydrolases, PBPs, or components of the Rod complex (10, 84); thus, the Mg^2+^ sensitivity of the ∆40 ∆*cwlO* strain seemed counterintuitive. Our experiments indicated that ∆40 ∆*cwlO* cells were sensitive to both Ca^2+^ and Mg^2+^; growth was inhibited by the addition of 10 mM MgCl_2_, 10 mM MgSO_4_, and 10 mM CaCl_2_ but not by the addition of 20 mM NaCl, suggesting that the growth inhibition was not due to changes in ionic strength or chloride ions (Fig. 6). We did observe growth inhibition due to ionic stress at far higher salt concentrations (500 mM NaCl). Notably, cells were not sensitive to equivalent osmotic stress (1 M sorbitol), indicating the sensitivity is to ionic stress, not osmotic stress.

As ∆*cwlO* mutants in the WT background were Mg^2+^ insensitive, we sought to identify which hydrolases caused cells to be sensitive to Mg^2+^ when they were removed. To find these hydrolases, we returned to intermediate strains used to construct the ∆40 strain, which are missing subsets of hydrolases. We transformed a *cwlO* knockout into these intermediate strains, then screened these crosses for the same small colony phenotype and the Mg^2+^ sensitivity that was seen in the ∆40 ∆*cwlO* strain. We identified two genes: *yabE* and *ydjM*. Notably, during the construction of the ∆40 strain, we also noticed that *yocH* seemed significant—at several intermediate verification steps, a WT copy of *yocH* had reintegrated itself during transformation with genomic DNA from single KO strains—we, therefore, used PCR product for all transformations after this. Furthermore, a *∆ydjM ∆yocH ∆cwlO* mutant was previously demonstrated to be sick, with short and sometimes anucleate cells (10). Because *yabE*, *ydjM*, and *yocH* have similar hydrolase domains, and because *yocH* and *ydjM* had been identified previously to be involved in a synthetic sick interaction with *cwlO,* we additionally tested whether the removal of *yocH* contributed to the ∆40 *∆cwlO* Mg^2+^ sensitivity phenotype and found that it did.

In total, we identified three genes, *yabE*, *ydjM*, and *yocH*, whose absence in a ∆*cwlO* background caused the Mg^2+^ sensitivity: A *∆yabE ∆ydjM ∆yocH ∆cwlO* strain showed a similar stress profile to ∆40 ∆*cwlO*, including sensitivity to MgCl_2_ and CaCl_2_ (Fig. 6 and 7A). *yabE*, *ydjM*, and *yocH* are three uncharacterized RlpA-like superfamily domain-containing proteins expressed during exponential growth. Like *lytE* and *cwlO, yocH* and *ydjM* are in the *walR* regulon, while *yabE* is regulated by *sigA* (Table 1). All are likely lytic transglycosylases: *yocH* has been shown to have lytic activity and has homology to the *E. coli* lytic transglycosylase *mltA* (40), and all three share a similar catalytic domain. Because *yabE*, *ydjM*, and *yocH* all contain a RlpA-like protein domain, we refer to these genes collectively as RLPAs, and to the triple deletion of all three genes as ∆RLPAs.

### LytE is inhibited by Mg^2+^ *in vitro* and *in vivo*, and RLPAs suppress Mg^2+^ lethality *in vivo*

Finally, we sought to identify the source of Mg^2+^ growth inhibition in the ∆RLPAs ∆*cwlO* background. Because LytE is essential in the absence of CwlO, we hypothesized that the sensitivity of the ∆40 ∆*cwlO* strain to Mg^2+^ (and, by extension, the sensitivity of the ∆RLPAs *∆cwlO* strain to Mg^2+^) could be explained by Mg^2+^ inhibition of LytE. To investigate this, we first characterized the response of ∆*cwlO* cells to the removal of LytE. We constructed an otherwise wild-type strain with *cwlO* knocked out and *lytE* under inducible control and monitored its growth by time-lapse phase-contrast microscopy. When *lytE* was induced, cell growth was normal (Movie S1). When *lytE* induction was removed, cell growth initially slowed, followed by a period of “stuttery” growth, where elongating cells intermittently shrank while showing accompanying fluctuations in their phase contrast signal (Movie S2). Ultimately, cells lysed about 1–2 doubling times after the removal of *lytE* induction, as previously observed (10, 12). Next, we performed the same imaging in the ∆RLPAs *∆cwlO* strain after the addition of 10 mM MgCl_2_ and observed the same “stuttery” phenotype, suggesting that LytE function might be inhibited by Mg^2+^ (Movie S4). Without the addition of Mg^2+^, cell growth of the ∆RLPAs *∆cwlO* strain was normal (Movie S3). In WT cells or *∆cwlO* cells, the presence of Mg^2+^ has no effect on cell viability or growth—growth is only inhibited in the absence of the RLPAs. Thus, the RLPAs appear to allow LytE to maintain its activity in the presence of Mg^2+^. This ∆RLPAs *∆cwlO* strain additionally had a similar environmental stress response profile as the ∆40 *∆cwlO* strain (Fig. 7A).

To test whether LytE activity is directly inhibited by Mg^2+^, we overexpressed and purified both full-length LytE and a truncated LytE protein with only its catalytic domain. *In vitro* activity assays with and without the addition of Mg^2+^ showed that indeed, LytE activity is inhibited by Mg^2+^ (Fig. 7B). Additionally, we reasoned that if the Mg^2+^-sensitivity phenotype was due to direct inhibition of LytE by Mg^2+^, increasing the levels of LytE should protect cells from death by increasing the total amount of LytE activity. Indeed, overexpression of LytE allowed the ∆RLPAs strain to survive in the presence of higher levels of Mg^2+^, although 100 mM MgCl_2_ still inhibited growth (Fig. 7C).

Thus, we conclude that LytE activity is inhibited by Mg^2+^ both *in vivo* and *in vitro*. Furthermore, our data indicate that the RLPAs allow LytE to maintain normal function in the presence of Mg^2+^, though the specific mechanism is unclear. Whether the RLPAs act directly or indirectly on LytE remains to be determined, but we anticipate that the RLPAs interact with and activate LytE similarly to what has been observed for the *Mycobacterium smegatis* hydrolases RipA and RpfB: RipA’s C-terminus (containing a NLPC/P60 domain like LytE) interacts with RpfB’s RlpA-like LTG domain (85), and RipA and RpfB have synergistic activity *in vitro* (86). By analogy, LytE’s catalytic NLPC/P60 domain may interact with the RlpA-like domains in YabE, YdjM, and YocH, leading to increased LytE activity, allowing LytE to continue to function in the presence of Mg^2+^. The ∆RLPAs ∆*cwlO* strain also has increased sensitivity to ionic stress and low temperatures, suggesting RLPAs might stimulate LytE activity under those conditions as well.

## DISCUSSION

Bacterial cell growth requires the action of PG hydrolases, but previous *in vivo* hydrolase studies have been impeded by their diversity and redundancy. We constructed and validated a *B. subtilis* strain lacking all hydrolases potentially involved in cell growth besides LytE and CwlO. These deletions constitute 40 genes in total, representing 10% of secreted proteins and 1% of all genes. The resulting ∆40 strain enables the investigation of given hydrolases and the cellular contexts in which they function, and in this work allowed several new discoveries regarding their sufficiency, regulation, and genetic interplay.

First, we found that the ∆40 strain is viable. This demonstrates that LytE and CwlO alone can function to expand the cell wall to allow cell growth. Furthermore, as single knockouts of LytE and CwlO in the ∆40 strain are viable and allow growth (albeit at somewhat reduced rates with some shape defects), this demonstrates that *B. subtilis* requires only one of these two hydrolases to grow.

Our minimal hydrolase strain allowed us to show that RlpA-like lytic transglycosylases enhance LytE activity *in vivo* and that this enhancement can be important for growth under conditions where LytE activity is inhibited, including the presence of divalent cations, ionic stress, and cold. Although the mechanism for LytE enhancement is unclear, we hypothesize that RlpAs stimulate LytE activity via a direct interaction, as has been observed for similar proteins in *Mycobacterium smegmatis* (86). Synthetic lethal or synthetic sick interactions are straightforward to identify and characterize in the ∆40 strain, giving a useful tool to interrogate genetic relationships between different hydrolases or between hydrolases and other genes of interest—such as those involved in cell wall synthesis.

Surprisingly, the growth rate of the ∆40 strain is only slightly impaired under standard lab conditions. What, then, is the function of these 40 hydrolases, and why does *B. subtilis* encode so many of them? This multitude of hydrolases likely arises from the fact that hydrolases are involved in other processes aside from cell growth, such as sporulation (4) and cell motility (87). Additionally, some hydrolases might only be needed under nutrient conditions not tested here, such as during phosphate limitation where teichoic acids are not produced, where cells may require hydrolases that are not regulated by teichoic acids (88 – 90). Finally, these other hydrolases may be important during non-exponential growth states such as during stationary phase, where the recycling of cell wall turnover products, lacking in the ∆40 strain, reduces cell lysis (91). Thus, a broader screen of the sensitivity of the ∆40 strain in different nutrient and environmental conditions will allow the determination of which hydrolases are useful for which conditions.

In summary, the ∆40 minimal hydrolase strain provides a powerful experimental background to investigate the function, regulation, and interplay of hydrolases, improving our understanding of precisely how these enzymes conduct their cellular tasks. In the future, individual hydrolases can be reintroduced into the ∆40 strain to investigate their specific activities in the absence of confounding contributions from the other 39 genes. Using the ∆40 strain, PG profiling can determine the biochemical activity of hydrolases. Uncovering synthetic genetic interactions between hydrolases and other genes of interest—now easy to do for all 40 hydrolases at once —will allow us to flesh out our understanding of bacterial cell growth. Understanding the function of cell wall hydrolases is essential for a complete understanding of how bacteria grow, and the ∆40 strain will allow rapid progress to this end.

## MATERIALS AND METHODS

### Strains, media, and growth conditions

Glycerol stocks stored at −80°C were streaked onto LB agar plates. For strain bSW61 (*lytE::pSpac-lytE, ∆cwlO*), these plates were additionally top spread with 1 mM IPTG. After incubation overnight at 37°C, colonies were inoculated into 1 mL media (the specific media used depended on the experiment, see figure legend for details) and grown on a roller at 37°C until they reached mid-exponential-phase growth (OD_600_ ~0.2). Cells were diluted 1:10 in prewarmed media and again grown until mid-exponential phase; this process was repeated until the start of the experiment. Alternately, a 1:10 dilution series of cells were grown overnight in media on a roller at 25°C. The next day, the culture whose OD_600_ was nearest to 0.2 was diluted 1:10 and grown in media at 37°C as above. S7_50_AA indicates S7_50_ media with added amino acids as in reference (92). CH indicates casein hydrolysate media, as in reference (93). LB indicates Luria Broth (Lennox) media for liquid media experiments and Luria Broth (Miller) for solid media (plates).

### Strain construction

The wild-type strain for this work was *B. subtilis* PY79. Strains used in this study are listed in Table S4. Constructs were created using Gibson assembly of PCR products. Linear Gibson assembly products were transformed into competent *B. subtilis*. Transformants were selected on LB plates containing the appropriate antibiotic. The resulting strains were verified by PCR. Constructs used in this study, as well as any plasmids used to create each construct, are listed in Table S4. Primers, along with strain construction details, are listed in Table S5. Resistance cassettes and promoters were amplified from purified plasmids (listed in Table S4), and all other fragments were amplified from WT gDNA.

To combine knockouts, the parent strain was transformed with PCR product containing the locus (homology arms + resistance cassette) or gDNA as indicated. All resistance cassettes used have loxP sites flanking the cassette, allowing Cre-based loopout using plasmid pDR244 (a gift from David Rudner) of the cassette to yield a markerless knockout. Removal of the plasmid was accomplished by shifting plates to 42°C, where it cannot be replicated due to a temperature-sensitive origin. Successful loopouts were confirmed via loss of antibiotic resistance.

### PHMMER search

We used pfamscan version 1.6 to search the *B. subtilis* 168 and PY79 proteomes for all pfam domains using default parameters: *e*-value: 0.01, significance *E*-values [hit]: 0.03, significance bit scores [sequence]: 25, significance bit scores [hit]: 22. We then filtered the list for domains of interest using the list of domains in Table S2, and identified putative membrane-bound/cytoplasmic proteins using UniProt (94).

### PG purification, HPLC conditions, and MS data analysis

PG purification was conducted as in reference (95), with an HF treatment step instead of HCl to remove teichoic acids and the addition of a protein digestion step. Cells were grown in a baffled flask to an OD_600_ of ~0.5 in 50 mL of CH media. Cells were mixed 50/50 with 50 mL of boiling 10% SDS and boiled for 15 min in a water bath, then pelleted at 5,000 × *g* and washed 5× with ddH_2_O. Cells were then resuspended in 2 mL DNase/RNase buffer (10 mM Tris pH 7.5, 2.5 mM MgCl_2_, and 0.5 mM CaCl_2_) with 20 µL DNase I (2000 units/mL) and 20 µL RNase A (20 mg/mL), then incubated overnight at 37°C and washed 3× with ddH_2_O to remove nucleic acids. Next, cells were resuspended in 2 mL Proteinase K buffer (10 mM Tris pH 7.5 and 1 mM CaCl_2_) with 20 µL Proteinase K (800 units/mL), incubated overnight at 45°C, and washed 3× with ddH_2_O to remove proteins. Next, cell walls were treated with 48% (vol/vol) hydrofluoric acid on ice for 24 h, then washed twice with 100 mM Tris pH 8 and four times with ddH_2_O. Then, the PG was resuspended in 12.5 mM NaHPO_4_ pH 5.5 with 5,000 units of mutanolysin and digested overnight (16 h) at 37°C on a roller to yield soluble muropeptides. Undigested material was pelleted by spinning at 16,000 × *g* for 5 min and the supernatant was transferred to a new tube. Soluble muropeptides were reduced with sodium borohydride (1 mg/mL) for 30 min and the reaction was stopped by adding 10 µL of 30% phosphoric acid. The pH was adjusted to 4–6 using NaOH, and the reduced soluble muropeptides were characterized by high-resolution LC-MS operating in both positive and negative modes. Soluble reduced muropeptides were separated on a Waters column with the following method: column temperature 52°C, flow rate 0.5 mL/min, linear gradient of solvent A [0.1% (wt/vol) formate] to 10% solvent B [acetonitrile + 0.1% (wt/vol) formate] over 80 min.

Feature detection was performed on the raw MS data using Dinosaur (96). Feature detection was done separately on both the positive and negative mode scans with default parameters. Feature data were analyzed using a custom MATLAB program, available at https://bitbucket.org/garnerlab/wilson_40_2020/. We first filtered feature data for charge <3. Next, we filtered for the top 10 features present during each scan. For each of these features, theoretical *m*/*z* values were compared with observed *m*/*z* with a cutoff of 10 ppm. We required that a compound be present on both the positive and negative scans and consolidated features matching the same compound within a retention time of 1 min. Finally, we filtered out compounds corresponding to in-source decay (loss of glucosamine without a change in retention time) and compounds present at less than 0.1% of all muropeptides. Retention times shown in Table S3 were analyzed manually.

### Growth rates

Cells were grown to an OD_600_ of ~0.3–0.5 on a roller drum at 37°C and diluted to an OD_600_ of ~0.05 in baffled flasks in a water bath shaker at 37°C. Samples were withdrawn at 5-min intervals and OD_600_ was measured in a plastic cuvette using a Biowave Cell Density Meter CO8000. T vs OD_600_ curves were fit to a single exponential (OD_600_ = Ae^BT^) to extract a growth rate (B).

### Autolysis rates

Cells were grown to an OD_600_ of ~0.3–0.5 on a roller drum at 37°C and diluted to an OD_600_ of ~0.025 into prewarmed CH in baffled flasks at 37°C. Once cells reached an OD_600_ of 0.5, sodium azide (75 mM final) or ampicillin (100 µg/mL final) was added to part of the culture and transferred to a prewarmed 96-well plate. OD_600_ was measured every 2 min for 24 h at 37°C using a BioTek Epoch 2 Microplate Spectrophotometer. The plate was shaken at maximum RPM in between measurements.

### Sporulation efficiency

Sporulation was induced by resuspension according to reference (93). Cells were grown to an OD_600_ of ~0.3–0.5 in CH media, pelleted, and resuspended in a resuspension medium. Sporulation efficiency was assessed by measuring the number of heat-resistant CFUs per mL of culture after 36 h. The cultures were heated to 80°C for 20 min and then plated. CFU counts were then done after 24 h of incubation at 37°C.

### Turnover rates

Turnover rates were measured as in reference (92) with some modifications; the method is summarized below. Cells were grown in S7_50_AA to an OD_600_ of ~0.3–0.5 on a roller drum at 37°C and diluted to an OD_600_ of ~0.05 in 3 mL of prewarmed S7_50_AA containing 1 μCi of ^3^H-N-acetylglucosamine [6-^3^H] (specific activity: 20 Ci/mmol, American Radiolabeled Chemicals, Inc., St. Louis, MI, USA) in 25 mm wide test tubes in a water bath shaker at 37°C. Cells were labeled for three generations (until OD_600_ ~ 0.4), then filtered, washed twice with prewarmed S7_50_AA, and resuspended in 25 mL of prewarmed S7_50_AA. Samples were withdrawn at 5-min intervals and OD_600_ was measured in a plastic cuvette using a Biowave Cell Density Meter CO8000. Samples were mixed 50:50 with ice-cold 10% (vol/vol) trichloroacetic (TCA) acid + 20 mM unlabeled GlcNAc, incubated on ice for 10 min, then filtered and washed. Filters were dried and resuspended in Ultima Gold LSC cocktail (PerkinElmer, Waltham, MA, USA), and radioactivity was measured using a scintillation counter (Tri-Carb 2100 TR, PerkinElmer). Decays/min vs OD_600_ plots were fit to a single exponential (DPM = Ae^BT^) to extract a turnover rate (B).

### Cell dimensions

Cells were grown to an OD_600_ of ~0.3–0.5 in a water bath shaker at 37°C. One milliliter of culture was stained with FM 5–95 and concentrated to 100 µL by centrifugation at 2,000 × *g* and resuspension. Five microliters of concentrated cells were spotted under 2% (wt/vol) agarose pads in CH containing 0.5 µg/mL FM 5–95. Images were collected on a Nikon Ti-E microscope using a Nikon CFI Plan Apo DM Lambda 100× Oil objective, 1.45 NA, phase ring Ph3 using an ORCA-Flash4.0 V2 sCMOS camera. Analysis was performed using Morphometrics v1.1 (74). Zero length or width cells were discarded, as well as any cells with width greater than the length. Outliers were removed using Graphpad Prism ROUT with default parameters (1%).

### Chain length

Cells were grown to an OD_600_ of ~0.3–0.5 on a roller drum at 37°C and diluted to an OD_600_ of ~0.025 into prewarmed CH in baffled flasks at 37°C. Once cells reached an OD_600_ of 0.5, 1–5 µL of culture was spotted under a prewarmed 2.5% (wt/vol) agarose pad in CH. A 10 × 10 image tile series was collected (~1.5 mm square). A custom MATLAB program was used to register and stitch the images together, and then chain length was measured manually with the assistance of a custom MATLAB program.

### Electron microscopy and cell wall thickness measurements

Electron microscopy was performed as in reference (97). Briefly, exponentially growing cells were fixed in 100 mM MOPS buffer pH 7 containing 2% (wt/vol) paraformaldehyde, 2.5% (wt/vol) gluteraldehyde, and 1% (vol/vol) dimethyl sulfoxide overnight at 4°C, washed, stained with 2% (wt/vol) osmium tetroxide in 100 mM MOPS for 1 h, washed, and stained overnight with 2% (wt/vol) uranyl acetate. The cells were then dehydrated and embedded in Embed 812 resin.

Serial ultrathin sections (80 nm) were cut with a Diatome diamond knife (EMS, PA) on a Leica Ultracut UCT (Leica Microsystems, Germany) and collected on 200-mesh thin-bar formvar carbon grids. Sections were imaged on a Hitachi HT7800 transmission electron microscope.

Images collected were segmented (inner cell wall, outer cell wall) using DeepCell (98), and cell wall thickness was measured using a custom MATLAB program available at https://bitbucket.org/garnerlab/wilson_40_2020/. Briefly, the distance between the inner and outer cell walls was measured every 10 nm along a user-defined line, and the mean of that measurement was taken to be the cell’s cell wall thickness.

### AFM imaging and cell wall thickness measurement

*B. subtilis* cells at mid-exponential phase were boiled rapidly to kill bacterial cells and inactivate any potential hydrolase activity. Cells were broken by French Press and FastPrep, then suspended in 5% (wt/vol) SDS and boiled for 25 min, and sacculi were collected by centrifugation at 20,000 *g* for 3 min. The resulting pellets were washed with distilled water to remove all traces of SDS, then re-suspended in Tris-HCl (50 mM, pH 7) containing 2 mg/mL pronase and incubated at 60°C for 90 min. The resulting sacculi were then re-suspended in LC-MS Chromasolv water for storage at −20°C.

Freshly cleaved mica discs were incubated with Cell-Tak {285 mL of 100 mM NaHCO_3_ (pH 8) then 10 µL of Cell-Tak [Corning, 5% (wt/vol) in acetic acid] and 5 µL of 1 M NaOH, covered and left for 20 min then washed five times with HPLC grade water} to ensure the attachment of sacculi on the glass surface. Sacculi were diluted in HPLC-grade water to appropriate concentration and dried onto mica using N_2_. These were further washed and dried with N_2_ again to remove any unattached sample.

All AFM data were taken on a JPK Nanowizard III in quantitative imaging mode. Samples were imaged in HPLC Grade water using a FastScanD cantilever (Bruker, Santa Barbara), nominal spring constant 0.25 N/m with a 256 × 256-pixel scan region, driven at ~167 Hz with a typical Z length of ~300 nm using peak interaction forces of 2–3 nN. Images were flattened to median of differences and first order planefit using Gwyddion.

### Spot dilution assay

Cells were grown to an OD_600_ of 0.5 and diluted 1:10 into 100 µL of LB media in a 96-well plate. A 1:10 serial dilution series was made, and 3 µL of each dilution was spotted onto the plate using a multichannel pipettor. The plates were allowed to dry and incubated in at 37°C or 42°C as indicated for 18 h. Plates incubated at 25°C or 18°C were left for additional time (24 and 48 h, respectively). Plates were photographed using a Canon SC1011 scanner with the lid open.

For the colony morphology assay in Fig. 1, this protocol was followed except that a colony of cells of each strain was simply resuspended in 100 µL of media using a toothpick (omitting the broth culture step).

### LytE purification

His-SUMO-tagged full length LytE missing the signal peptide (26-355) and His-SUMO-tagged truncated LytE missing its 3× LysM domains (185-255) were overexpressed and purified under denaturing conditions from *E. coli* BL21. Three liters of LB Amp (100 µg/mL) culture were grown to an OD_600_ of 0.7 and overexpression was induced with 1 mM IPTG. Cultures were induced for 6 h, then harvested by centrifugation. Pellets were frozen at −80°C for storage. For purification, pellets were thawed and resuspended in lysis buffer [100 mM sodium phosphate, 10 mM Tris, 10 mM imidazole, 1% (vol/vol) Triton, 8M Urea, pH 8]. Cells were lysed by sonication, and cell debris was removed by centrifugation. A His column was equilibrated in lysis buffer (3 mL bed volume). Clarified lysate was passed through the His column. The bound protein was washed once with 50 mM HEPES, 150 mM NaCl, 20 mM imidazole, 1% (vol/vol) Triton, 8 M Urea, pH 8. Proteins were renatured on the column in 50 mM HEPES, pH 8 with 1% (vol/vol) Triton and 20 mM imidazole with a steady reduction of urea concentration (8 M, 6 M, 4 M, 2 M, 1 M, and 0 M) and increasing NaCl concentration (150 mM, 237.5 mM, 325 mM, 412.5 mM, 456.25 mM, and 500 mM). Refolded proteins were eluted from the column by increasing the imidazole concentration to 250 mM. dithiothreitol (DTT) was added to 1 mM to all fractions. Fractions containing the target protein were pooled and dialyzed overnight at 4°C in cleavage buffer (50 mM HEPES pH 8, 500 mM NaCl, and 2 mM DTT) with the addition of purified Ulp1 to cleave the 6His-SUMO tag. The buffer was exchanged once and further dialyzed for several hours. A new column was equilibrated in cleavage buffer without DTT and the pooled, cleaved protein was run through the column to remove the 6His-SUMO tag. Fractions containing cleaved protein were pooled and concentrated to a volume of 2 mL, then stored in dialysis in cleavage buffer. Activity tests were performed using purified PG in cleavage buffer plus 0.5% (wt/vol) Pluronic F-108 and PG from Sigma to an OD_600_ of 0.25 at 37°C. OD_600_ was measured every 2 min for 24 h using a BioTek Epoch 2 Microplate Spectrophotometer. The plate was shaken at maximum RPM in between measurements.

## Data Availability

All custom software used in this work is available at https://bitbucket.org/garnerlab/wilson_40_2020/. Raw HPLC-MS data for PG profiling experiments are available from MassIVE MSV000086886 (doi:10.25345/C5R21D). Raw and error corrected sequencing reads for whole genome sequencing are available at BioProject PRJNA702153.

## SUPPLEMENTAL MATERIAL

Supplemental figures and tables mbio.01760-23-s0001.docx Tables S1 and S2; captions for Tables S3 to S5; Fig. S1 to S3; legends for Movies S1 to S4.

Table S3 mbio.01760-23-s0002.xlsx Detailed PG profiling results.

Table S4 mbio.01760-23-s0003.xlsx Strains and plasmids used in this study.

Table S5 mbio.01760-23-s0004.xlsx Strain construction details and primer sequences.

Movie S1 mbio.01760-23-s0005.mov Growth of inducible *lytE, ∆cwlO* strain in the presence of inducer. Cells were spotted under an agarose pad containing media with inducer (CH + 250 µM IPTG) and imaged using phase-contrast microscopy. Frames are 1 minute apart. Strains used: bSW61, *lytE::pSpac-lytE*.

Movie S2 mbio.01760-23-s0006.mov ‘Stuttery’ growth before lysis of inducible *lytE, ∆cwlO* strain upon removal of inducer. Cells were spotted under an agarose pad containing media without inducer (CH) and imaged using phase-contrast microscopy. Frames are 1 minute apart. Strains used: bSW61, *lytE::pSpac-lytE*.

Movie S3 mbio.01760-23-s0007.mov Normal growth of WT cells before and after addition of Mg^2+^. Cells were loaded into a CellASIC BO4A plate in CH media and imaged using phase-contrast microscopy. At frame 18, media was exchanged for the same media plus 20 mM Mg^2+^ (indicated by label in upper left hand corner.) Frames are 2 minutes apart. Strains used: WT, PY79.

Movie S4 mbio.01760-23-s0008.mov ‘Stuttery’ growth of ∆RLPAs *∆cwlO* strain only after addition of Mg^2+^. Cells were loaded into a CellASIC BO4A plate in CH media and imaged using phase-contrast microscopy. At frame 18, media was exchanged for the same media plus 20 mM Mg^2+^ (indicated by label in upper left hand corner.) Frames are 2 minutes apart. Strains used: bSW490, ∆RLPAs *∆cwlO*.

ASM does not own the copyrights to Supplemental Material that may be linked to, or accessed through, an article. The authors have granted ASM a non-exclusive, world-wide license to publish the Supplemental Material files. Please contact the corresponding author directly for reuse.
