# Supplementary material for: An exhaustive multiple knockout approach to understanding cell wall hydrolase function in Bacillus subtilis
Source: mBio. 2023 Sep 28;14(5):e01760-23. doi: 10.1128/mbio.01760-23 (PMC10653849; doi:10.1128/mbio.01760-23)
Supplement: Supplemental figures and tables — Tables S1 and S2; captions for Tables S3 to S5; Fig. S1 to S3; legends for Movies S1 to S4. [file mbio.01760-23-s0001.docx]

**Supplemental Table 1: 8 SNPs are present in the ∆40 strain, all in pathways unrelated to exponential phase cell wall synthesis.**

| Change | Codon Change | AA Change | Gene Name | CDS Name | CDS Position | Coverage | Protein Effect | Locus |
| --- | --- | --- | --- | --- | --- | --- | --- | --- |
| T -> A | TCA -> ACA | S -> T | *mfd* | transcription-repair coupling factor CDS | 1216 | 95 | Substitution | BSU00550 |
| T -> G | CAA -> CCA | Q -> P | *spsB* | spore coat polysaccharide biosynthesis protein SpsB CDS | 1160 | 61 | Substitution | BSU37900 |
| T -> C | ATC -> ACC | I -> T | *pucL* | uric acid degradation bifunctional protein PucL CDS | 194 | 46 | Substitution | BSU32450 |
| G -> A | CCA -> CTA | P -> L | *panD* | aspartate 1-decarboxylase CDS | 365 | 31 | Substitution | BSU22410 |
| C -> A | TGG -> TGT | W -> C | *dhbF* | non-ribosomal peptide synthetase CDS | 3732 | 9 | Substitution | BSU31960 |
| T -> C | GCA -> GCG |  | *opuBA* | choline transport ATP-binding protein OpuBA CDS | 447 | 58 | None | BSU33730 |
| G -> T | CGG -> CGT |  | *ykvU* | sporulation protein YkvU CDS | 603 | 37 | None | BSU13830 |
| G -> A | GCC -> GCT |  | *czcD* | H+/K+ antiporter CDS | 504 | 30 | None | BSU26650 |

Whole-genome sequencing (Illumina, paired-end) was performed on the ∆40 strain. Libraries were prepared using an Illumina Nextera XT kit. QC was performed using qPCR and TapeStation, and sequencing was performed using an Illumina NextSeq 500. 8 SNPs were detected at >8x coverage at 100% variant frequency, listed here. Strains used: bSW431, ∆40.

**Supplemental Table 2: List of PFAM domains with cell wall hydrolase activity included in our search.**

| Pfam accession | Domain name | Example protein | Reference |
| --- | --- | --- | --- |
| PF01510 | Amidase_2 | XlyA | (1) |
| PF01520 | Amidase_3 | LytC | (1) |
| [PF12671](http://pfam.xfam.org/family/PF12671) | Amidase_6 | YhbB | (3) |
| [PF07454](http://pfam.xfam.org/family/PF07454) | SpoIIP | SpoIIP | (4) |
| PF00144 | Beta-lactamase | PbpX | (5) |
| PF01915 | Glyco_hydro_3_C | NagZ | (2) |
| PF00704 | Glyco_hydro_18 | SleL | (6) |
| PF01832 | Glucosaminidase | LytD | (1) |
| PF06725 | 3D | YocH | (7) |
| PF07486 | Hydrolase_2 | CwlJ | (1) |
| [PF01464](http://pfam.xfam.org/family/PF01464) | SLT | CwlP | (1) |
| PF03330 | DPBB_1 | YdjM | (9) |
| PF08486 | SpoIID | SpoIID | (10) |
| PF00877 | NLPC_P60 | LytE | (1) |
| PF00246 | Peptidase_M14 | YqgT | (2) |
| PF01551 | Peptidase_M23 | LytH | (2) |
| PF05708 | Peptidase_C92 | YycO | (2) |
| PF13539 | Peptidase_M15_4 | CwlK | (1) |
| PF05382 | Amidase_5 |  | (2) |
| PF00933 | Glyco_hydro_3 | NagZ | (2) |
| PF00062 | Lys |  | (2) |
| PF00959 | Phage_lysozyme | | (2) |
| PF01183 | Glyco_hydro_25 | | (2) |
| PF05257 | CHAP | CwlO | (2) |
| PF00877 | NLPC_P60 | LytE | (2) |
| PF03411 | Peptidase_M74 | | (2) |
| PF14718 | SLT_L | EcSlt70 | (8) |
| PF03562 | MltA | EcMltA | (8) |
| [PF13406](http://pfam.xfam.org/family/PF13406) | SLT_2 | EcMltB | (8) |
| PF11873 | Mltc_N | EcMltC | (8) |
| [PF11741](http://pfam.xfam.org/family/PF11741) | AMIN | EcAmiC | (8) |
| [PF01427](http://pfam.xfam.org/family/PF01427) | Peptidase_M15 | CwlK | (2) |
| PF13702 | Lysozyme_like | YddH | (1) |
| PF02618 | YceG | EcMltG | (11) |

Hydrolase domains were collated from 3 major reviews: (1) Smith et al. 2000, (2) Vermassen et al. 2019, and (8) ﻿van Heijenoort et al. 2011, as well as other sources as indicated.

**Supplemental Table 3: Detailed PG profiling analysis results**

Full name, retention time (with standard deviation), percent of total muropeptides (with standard deviation), as well as one-letter-code labels and associated hydrolases for each detected muropeptide present at more than 0.1% abundance in at least one condition is given for WT, ∆40 and ∆40 ∆mltG strains. Strains used: PY79, WT; bSW431, ∆40; bSW537, ∆40 *∆mltG*.

**Supplemental Table 4: Strains and plasmids used in this study**

Detailed genotypes, descriptions, and sources for the strains and plasmids used in this study are given.

**Supplemental Table 5: Strain construction details and primer sequences**

Strain names and genotypes for all intermediate strains used to construct the final strains are given. Primer sequences for both strain construction and verification are given. In general, ‘up’ and ‘down’ primers were used to amplify homology arms, ‘long’ verification primers were used to verify single knockout strains, and ‘short’ verification primers were used to verify successful loopouts in the ∆40 strain construction process.

**Supplemental Figure 1: Sequencing coverage patterns in the ∆40 strain**


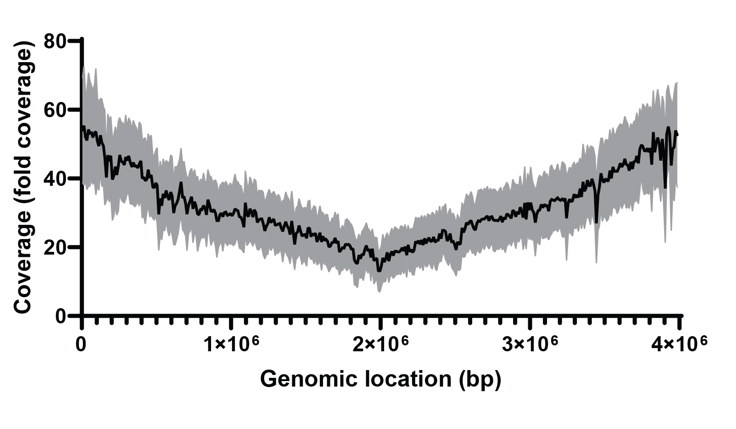


Sequencing (described in Methods and Supplemental Table 1) coverage results for the ∆40 strain. Repeat regions were excluded from coverage analysis. Mean (black line) and standard deviation (grey shading) of coverage for 1kbp regions was computed. Strains used: bSW431, ∆40.

**Supplemental Figure 2: EIC traces for several sample PG species.**


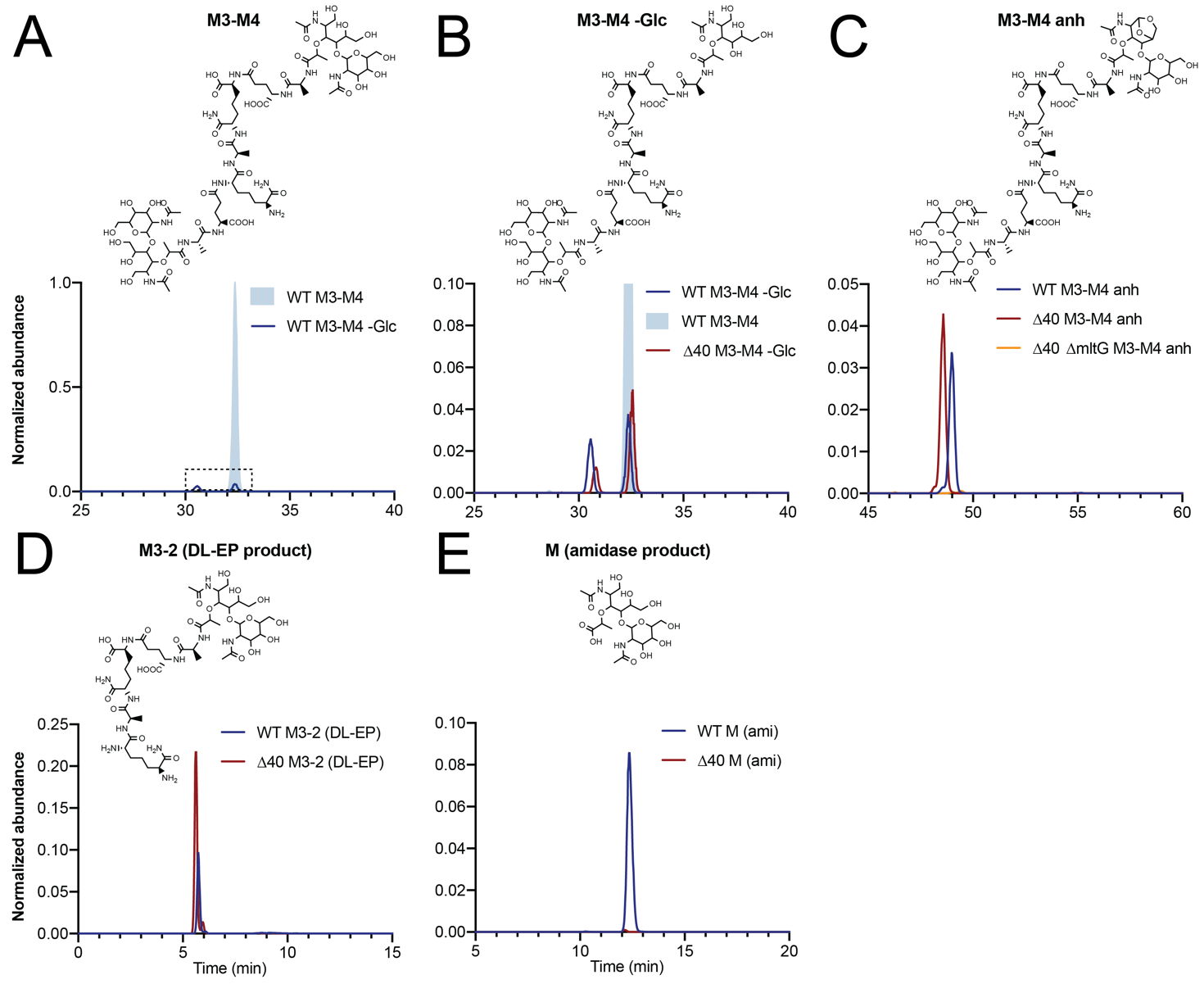


Extracted ion current (EIC) traces for several representative PG species in both WT, ∆40, and ∆40 ∆mltG strains. (A) Disaccharide tripeptide disaccharide tetrapeptide with 2 amidations (M3-M4, m/z = 896.90712, blue shaded) and disaccharide tripeptide disaccharide tetrapeptide with 2 amidations missing a glucosamine (M3-M4 -Glc, m/z = 795.36744, dark blue line) are shown. The maximum abundance of M3-M4 was used to normalize the abundance of all other species shown in all plots. (B) Zoomed view of the plot shown in A with the addition of data from the ∆40 strain. M3-M4 -Glc in the blue shaded region (same retention time as M3-M4) likely represents in-source decay of the M3-M4 PG species, while the M3-M4 -Glc with a retention time around 30 mins likely represents glucosamindase product. (C) Anhydrodisaccharide tripeptide disaccharide tetrapeptide with 2 amidations (m/z = 886.89402) abundance is shown for WT, ∆40, and ∆40 ∆mltG strains. (D) Disaccharide tripeptide dipeptide with 2 amidations (m/z = 556.76951) abundance is shown for WT and ∆40 strains. (E) Disaccharide (m/z = 499.21336) abundance is shown for WT and ∆40 strains. Strains used: PY79, WT; bSW431, ∆40; bSW537, ∆40 *∆mltG*.**
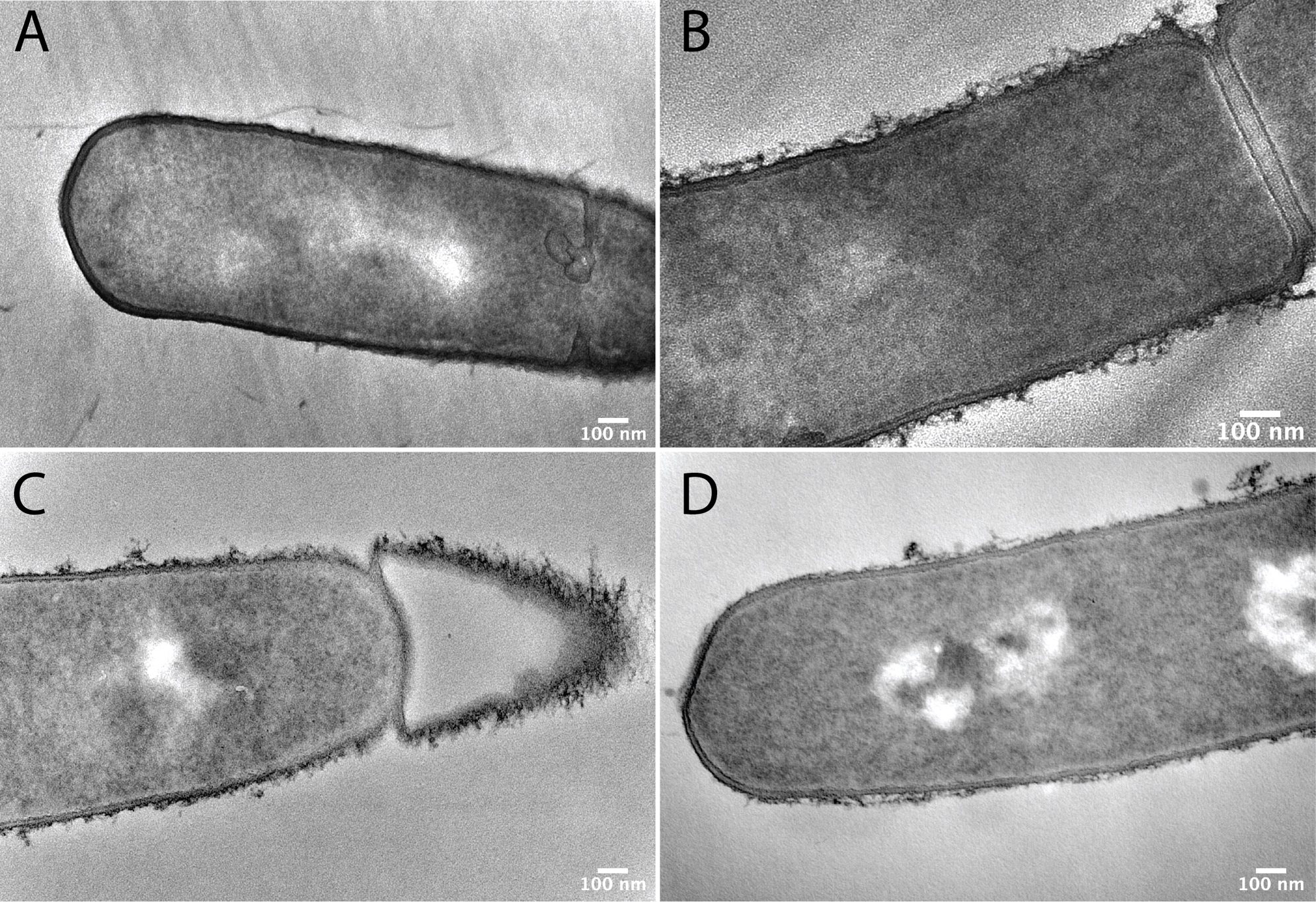
**

**Supplemental Figure 3: Representative image of cells via TEM.**

**A: Whole cell image of PY79 (WT)** from the dataset analyzed in Figure 4B. Strains used: PY79, WT.

**B: Whole cell image of ∆40** from the dataset analyzed in Figure 4B. Strains used: bSW431, ∆40.

**C: Whole cell image of ∆40 ∆lytE,** prepared as in Figure 4B (details in Methods). Strains used: bSW435, ∆40 ∆lytE.

**D: Whole cell image of ∆40 ∆cwlO,** prepared as in Figure 4B (details in Methods). Strains used: bSW433, ∆40 ∆cwlO.

**Supplemental Movie 1: Growth of inducible *lytE, ∆cwlO* strain in the presence of inducer.** Cells were spotted under an agarose pad containing media with inducer (CH + 250 µM IPTG) and imaged using phase-contrast microscopy. Frames are 1 minute apart. Strains used: bSW61, *lytE::pSpac-lytE*.

**Supplemental Movie 2: ‘Stuttery’ growth before lysis of inducible *lytE, ∆cwlO* strain upon removal of inducer.** Cells were spotted under an agarose pad containing media without inducer (CH) and imaged using phase-contrast microscopy. Frames are 1 minute apart. Strains used: bSW61, *lytE::pSpac-lytE*.

**Supplemental Movie 3: Normal growth of WT cells before and after addition of Mg^2+^.** Cells were loaded into a CellASIC BO4A plate in CH media and imaged using phase-contrast microscopy. At frame 18, media was exchanged for the same media plus 20 mM Mg^2+^ (indicated by label in upper left hand corner.) Frames are 2 minutes apart. Strains used: WT, PY79.

**Supplemental Movie 4: ‘Stuttery’ growth of ∆RLPAs *∆cwlO* strain only after addition of Mg^2+^** Cells were loaded into a CellASIC BO4A plate in CH media and imaged using phase-contrast microscopy. At frame 18, media was exchanged for the same media plus 20 mM Mg^2+^ (indicated by label in upper left hand corner.) Frames are 2 minutes apart. Strains used: bSW490, ∆RLPAs *∆cwlO*.

**SUPPLEMENTAL REFERENCES**

1. Smith TJ, Blackman S a., Foster SJ. 2000. Autolysins of Bacillus subtilis: Multiple enzymes with multiple functions. Microbiology 146:249–262.

2. Vermassen A, Leroy S, Talon R, Provot C, Popowska M, Desvaux M. 2019. Cell wall hydrolases in bacteria: Insight on the diversity of cell wall amidases, glycosidases and peptidases toward peptidoglycan. Front Microbiol 10.

3. Eichenberger P, Jensen ST, Conlon EM, Van Ooij C, Silvaggi J, González-Pastor JE, Fujita M, Ben-Yehuda S, Stragier P, Liu JS, Losick R. 2003. The σE regulon and the identification of additional sporulation genes in Bacillus subtilis. J Mol Biol 327:945–972.

4. Chastanet A, Losick R. 2007. Engulfment during sporulation in Bacillus subtilis is governed by a multi-protein complex containing tandemly acting autolysins. Mol Microbiol 64:139–152.

5. Palomino MM, Sanchez-Rivas C, Ruzal SM. 2009. High salt stress in Bacillus subtilis: involvement of PBP4* as a peptidoglycan hydrolase. Res Microbiol 160:117–124.

6. Lambert EA, Popham DL. 2008. The Bacillus anthracis SleL (YaaH) protein is an N-Acetylglucosaminidase involved in spore cortex depolymerization. J Bacteriol 190:7601–7607.

7. Shah IM, Dworkin J. 2010. Induction and regulation of a secreted peptidoglycan hydrolase by a membrane Ser/Thr kinase that detects muropeptides. Mol Microbiol 75:1232–1243.

8. van Heijenoort J. 2011. Peptidoglycan hydrolases of Escherichia coli. Microbiol Mol Biol Rev 75:636–663.

9. Jorgenson MA, Chen Y, Yahashiri A, Popham DL, Weiss DS. 2014. The bacterial septal ring protein RlpA is a lytic transglycosylase that contributes to rod shape and daughter cell separation in Pseudomonas aeruginosa. Mol Microbiol 93:113–128.

10. Abanes-De Mello A, Sun YL, Aung S, Pogliano K. 2002. A cytoskeleton-like role for the bacterial cell wall during engulfment of the Bacillus subtilis forespore. Genes Dev 16:3253–3264.

11. Yunck R, Cho H, Bernhardt TG. 2016. Identification of MltG as a potential terminase for peptidoglycan polymerization in bacteria. Mol Microbiol 99:700–718.
